# Cell cycle regulation has shaped replication origins in budding yeast

**DOI:** 10.1038/s41594-025-01591-9

**Published:** 2025-06-30

**Authors:** Chew Theng Lim, Thomas C. R. Miller, Kang Wei Tan, Saurabh Talele, Anne Early, Philip East, Humberto Sánchez, Nynke H. Dekker, Alessandro Costa, John F. X. Diffley

**Affiliations:** 1 Chromosome Replication Laboratory, The Francis Crick Institute, London, UK; 2 Macromolecular Machines Laboratory, The Francis Crick Institute, London, UK; 3 Center for Chromosome Stability, Department of Cellular and Molecular Medicine, University of Copenhagen, Copenhagen, Denmark; 4 Department of Bionanoscience, Kavli Institute of Nanoscience, Delft University of Technology, Delft, The Netherlands; 5 Department of Physics and Kavli Institute of Nanoscience Discovery, University of Oxford, Oxford, United Kingdom; 6 Bioinformatics & Biostatistics, The Francis Crick Institute, London, UK

**Keywords:** DNA, Cryoelectron microscopy, DNA replication, Origin selection

## Abstract

Eukaryotic DNA replication initiates from genomic loci known as origins. At budding yeast origins like ARS1, a double hexamer (DH) of the MCM replicative helicase is assembled by origin recognition complex (ORC), Cdc6 and Cdt1 by sequential hexamer loading from two opposed ORC binding sites. Cyclin-dependent kinase (CDK) inhibits DH assembly, which prevents re-replication by restricting helicase loading to the G1 phase. Here, we show that an intrinsically disordered region (IDR) in the Orc2 subunit promotes interaction between ORC and the first loaded, closed-ring MCM hexamer (the MCM–ORC (MO) intermediate). CDK-dependent phosphorylation of this IDR blocks MO formation and DH assembly. We show that MO stabilizes ORC at lower-affinity binding sites required for second hexamer loading. Origins comprising two high-affinity ORC sites can assemble DH efficiently without MO by independently loading single hexamers. Strikingly, these origins escape CDK inhibition in vitro and in vivo. Our work reveals mechanistic plasticity in MCM loading with implications for understanding how CDK regulation has shaped yeast origin evolution and how natural, strong origins might escape cell cycle regulation. We also identify key steps common to loading pathways, with implications for understanding how MCM is loaded in other eukaryotes.

## Abstract (web summary)

Licensing of eukaryotic origins of replication with MCM double hexamers (DHs) can occur through distinct pathways. Here, Lim et al. show that in yeast, cell cycle-dependent regulation of DH formation by CDK and origin structure have co-evolved.

## Main

The initiation of eukaryotic DNA replication is separated into two temporally distinct steps that ensure no region of the genome is replicated more than once^1–3^. During the G1 phase, a pair of hexameric MCM helicases is loaded at each replication origin around double-stranded DNA in the form of a head-to-head MCM-DH. During S-phase, each DH is converted into two active CMG (Cdc45–MCM–GINS) helicases, thereby initiating bidirectional DNA replication.

**Fig. 1:**
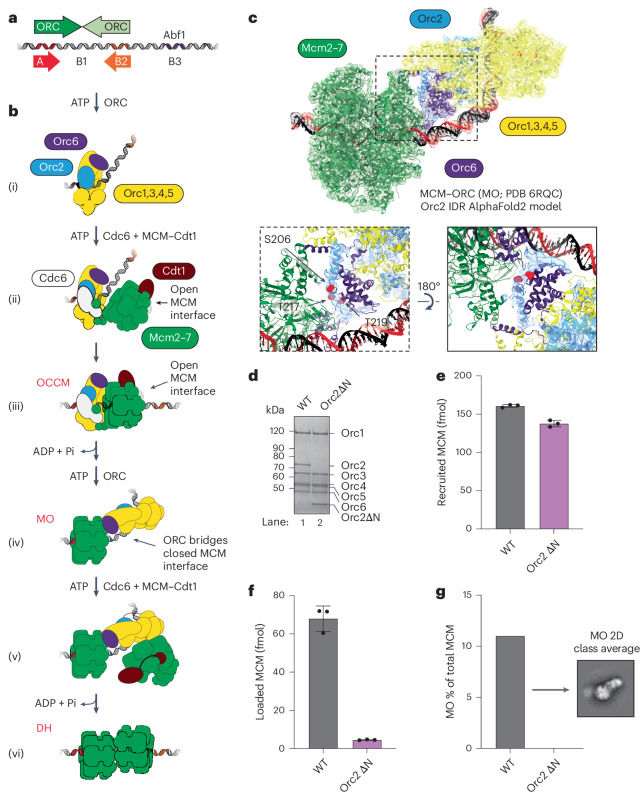
The Orc2 IDR is a key element of the MCM–ORC interface. **a**, Architecture of the canonical ARS1 origin of replication, with high-affinity (dark green) and low-affinity (light green) ORC binding sites. **b**, Schematic of the DH formation pathway: (i) ORC binds an A/B1 element and bends the DNA. (ii) ORC recruits a first MCM hexamer and (iii) threads DNA through the open Mcm2–5 gate, forming an OCCM intermediate. (iv) ATP hydrolysis promotes OCCM disassembly and first MCM loading. ORC is released from A/B1, and an ORC binds to both the B2 and the N-terminal face of the first loaded MCM, forming an MCM–ORC (‘MO’) complex. ORC recruits (v) and then loads (vi) a second MCM onto the origin, forming a DH. **c**, Cryo-EM structure of the MCM–ORC intermediate (PDB 9I3I, EMD-4980 (ref. ^11^)), including the Orc2 IDR (amino acids 190–231) modeled by AlphaFold2 (ref. ^14^). The Orc2 IDR, including CDK phosphorylation sites S206, T217 and T219, occupies previously unassigned EM density at the center of the MCM–ORC interface (see dotted insert on left and a 180° rotated view on the right). The Orc2 EM density has been isolated from a density-modified (EMready^45^) map of ORC obtained from the multibody refinement of the MO^11^. **d**, Orc2∆N is stably incorporated into the ORC complex. **e**, DNA-pulldown assays show Orc2∆N recruits luciferase-tagged MCM to ARS1 in ATPγS with comparable efficiency to WT ORC. **f**, Orc2∆N is compromised in MCM loading on ARS1 in ATP. The data in (**e**) and (**f**) are plotted as means of three technical replicates; error bars, s.d. **g**, MCM loading assays visualized by negative-stain EM show that Orc2∆N fails to form MO on ARS1. Quantification of micrographs, total MCM and MO in negative-stain EM 2D classification data: WT (156, 6,159, 680); Orc2∆N (167, 5,733, not detected). Source data

Budding yeast DNA replication origins generally contain a high-affinity ORC binding site flanked by one or more low-affinity sites in the opposite orientation^4–6^. At ARS1 (Fig. 1a), the bipartite high-affinity site comprises the A and B1 elements (A/B1), and a lower-affinity site corresponds to the B2 element^6–8^. Both the A and B2 elements contain the extended ARS consensus sequence (EACS); the A element is a closer match to the EACS than B2. DH assembly at ARS1 occurs by sequential loading of two MCM hexamers (outlined in Fig. 1b)^5,9,10^. After loading of the first hexamer (Fig. 1b(i–iii)), ORC releases from A/B1. ORC then binds both B2 and the amino-terminal domain of the first loaded MCM, generating the ‘MO’ complex (Fig. 1b(iv))^11^. The same ORC that was bound to A/B1 can ‘flip’ and bind B2 (ref. ^12^); alternatively, a second ORC molecule can bind B2 and engage the first recruited MCM (ref. ^5^). In MO, the MCM ring is closed and MCM subunits are primarily in a post-hydrolysis (ADP-bound) state, very similar to the DH^11^. Given that this second ORC binding event also induces a DNA bend, the face of ORC that promotes initial MCM recruitment and ORC–Cdc6–Cdt1–MCM (OCCM) formation is available in MO to receive the second MCM–Cdt1 without steric interference (Fig. 1b(v)). MO is thus poised to be a key intermediate in DH assembly (Fig. 1b(vi)); nonetheless, the exact mechanism of MO formation, its function and its importance in DH assembly remain unclear. Moreover, whether all DH assembly proceeds via MO is unknown.

CDKs have a crucial role in ensuring that replication occurs only once per cell cycle by inhibiting DH assembly outside of the G1 phase. They do so by promoting proteasome-dependent degradation of Cdc6 and nuclear exclusion of MCM–Cdt1. They also inhibit DH assembly directly by phosphorylation of the ORC subunits Orc2 and Orc6 (refs. ^1–3^). Single-molecule experiments have recently indicated that Orc6 phosphorylation promotes premature release of MCM–Cdt1, while stable MCM ring closure around DNA and MO formation do not occur efficiently after phosphorylation of Orc2 or Orc6 (ref. ^13^).

## Results

### MO is crucial for MCM loading at ARS1

**Extended Data Fig. 1:**
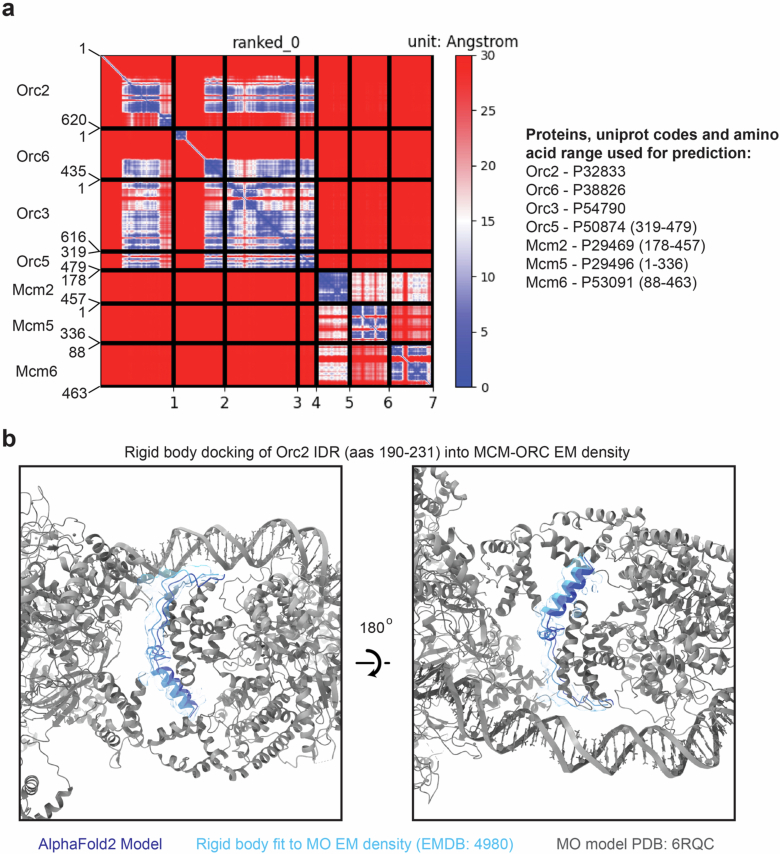
AlphaFold2 places the Orc2 IDR at the MCM-ORC (MO) interface. **a**. Predicted aligned error (PAE) plot for the AlphaFold2 model of the MO interface. The structure prediction was based on proteins present at the MO interface in the published structure (PDB: 6RQC ref. ^11^). **b**. Following alignment of Orc3 (aa 270-419) in the top ranked AlphaFold2 model with the respective region of Orc3 in the MO structure, the predicted Orc2 IDR (aas 190-231) was fit as a rigid body into previously unassigned EM density in the MO map. Here, the density displayed has been isolated from a density modified (EMready^45^) map of ORC obtained from the multibody refinement of the MO (EMDB: 4980 ref. ^11^). The prediction places the Orc2 IDR as wrapping around the Orc6 TFIIB-B domain at the center of the MO interface.

We first sought to understand the importance and role of MO in DH assembly by identifying an ORC mutant that cannot form MO. We have previously shown that an N-terminal deletion of the Orc6 subunit of ORC (Orc6∆119) reduced both MO and DH formation by approximately 50%, consistent with the idea that MO is a precursor of DH^11^; however, this was a relatively modest effect, and we wanted to generate a mutant with more complete loss of MO formation. Orc2 contains a long (235 amino acid) N-terminal extension that is predicted by AlphaFold^14^ to be intrinsically disordered. The Orc2 IDR has not been resolved in any ORC structure determined experimentally, including MO. A region of the Orc2 IDR was tentatively assigned as wrapping around Orc6 in a cryogenic electron microscopy (cryo-EM) density map of DNA-bound ORC^15^, albeit at a resolution that prevented assignment of the amino acid sequence and inclusion of this region in the entry deposited in the Protein Data Bank (PDB 5ZR1 (ref. ^15^)). Using AlphaFold2 multimer^16^, we identified a region of the Orc2 IDR docking on the carboxy-terminal domain of Orc6 with high confidence (Fig. 1c and Extended Data Fig. 1), consistent with the density seen in that previous work^15^. Notably, overlaying our AlphaFold prediction with our previously determined structure of the MO intermediate places the region of the Orc2 N-terminal IDR (amino acids 190–231) into a contiguous stretch of density at the core of the MCM–ORC interface, suggesting that it may have a key role in MO formation (Fig. 1c). Deletion of this IDR from Orc2 did not affect the ability of Orc2 to form a stable complex with the other ORC subunits (Fig. 1d). ORC complexes containing this deletion (Orc2∆N) recruited MCM normally in ATPγS (Fig. 1e). However, Orc2∆N loaded MCM at a level only 7% that of wild-type (WT) ORC (Fig. 1f). To visualize the effect of Orc2 IDR truncation on DH formation directly, we performed MCM loading assays on a 454 bp linear DNA template containing ARS1 flanked by nucleosomes and imaged the reactions by negative-stain EM. Although MO was readily detectable with WT ORC in ATP, consistent with previous work^11^, MO was not detected in reactions with Orc2∆N (Fig. 1g). These experiments show that the Orc2 IDR is essential for forming the MO intermediate, and that the inability to form MO compromises DH assembly, indicating that MO is a crucial intermediate in the assembly of the DH at ARS1. Small amounts of DH assembly by Orc2∆N suggest the existence of an inefficient, MO-independent DH assembly pathway at ARS1.

### MO is required to stabilize ORC at weak binding sites

**Fig. 2:**
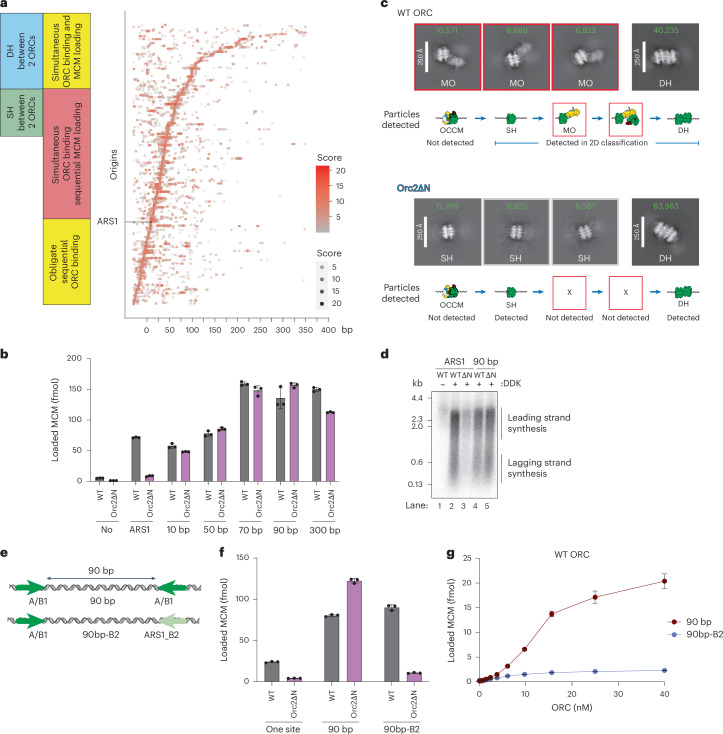
The MO promotes MCM loading by stabilizing ORC at low-affinity binding sites. **a**, Matches to the EACS were identified using a position weight matrix (PWM) as previously described^5^. Origins are arranged according to the distance between the center of the best match to the EACS and the center of the best B2-like EACS by PWM in the opposite orientation. PWM scores are indicated by the size and color of dots, as indicated in the figure. **b**, Orc2∆N loads MCMs onto origins containing two high-affinity inverted ORC binding sites spaced ≥10 bp apart with comparable affinity to WT ORC. The data are plotted as means of three technical replicates; error bars, s.d. **c**, Cryo-EM 2D classification of early time-point MCM loading assays showing that Orc2∆N cannot form MO intermediates on 90 bp origins containing symmetrical high-affinity ORC binding sites. Green numbers indicate the number of particles contributing to each class average. Total micrographs analyzed (WT, 18,156; Orc2∆N, 18,414); total particles containing single MCM helicases (WT, 188,324; Orc2∆N, 244,701); and total particles containing MCM-DHs (WT, 149,543; Orc2∆N, 137,314). The top three (SH-containing) or one (DH) classes are shown; the full classification is shown in Extended Data Fig. 3. **d**, MCMs loaded onto 90 bp origins by WT ORC and Orc2∆N are replication competent. **e**, Schematic of synthetic origins containing inverted high-affinity ORC binding sites spaced 90 bp apart (top), or with one high-affinity site replaced by the ARS1 B2 element (bottom). **f**, MCM loading on 90 bp origins by Orc2∆N is dependent on two high-affinity ORC binding sites. The data are plotted as means of three technical replicates; error bars, s.d. **g**, MCM loading by WT ORC, showing two DNA-bound ORCs load MCM more efficiently than one ORC at various concentrations. ORC was preincubated with DNA beads and washed twice with LSW + 150 mM NaCl buffer. Cdc6 and MCM–Cdt1 were then added to the reaction and incubated for another 5 min. The data are plotted as means of three technical replicates; error bars, s.d. Source data

**Extended Data Fig. 2:**
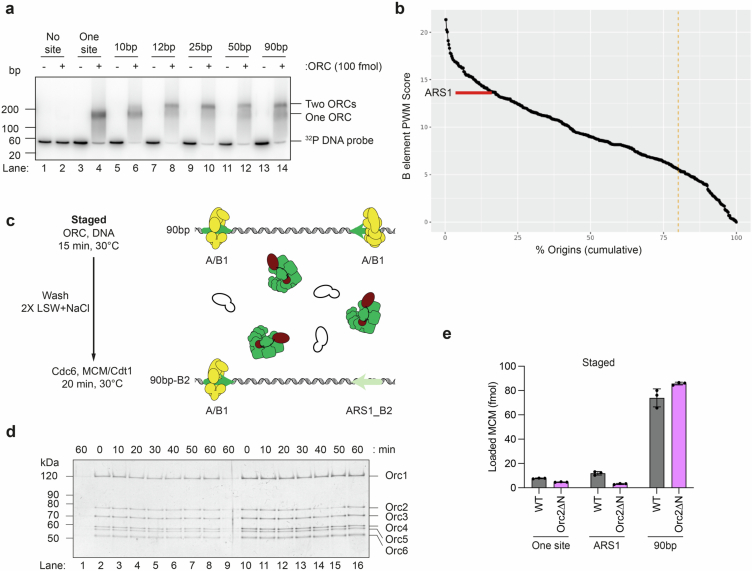
The formation of MO stabilizes ORC binding at weak binding sites. **a**. EMSA of ORC binding at different distances on synthetic templates. The 10 bp distance is similar to ARS1, which can only bind a single ORC. Two ORCs can bind simultaneously on origins with a spacing of ≥12 bp. **b**. Approximately 15% of origins have a better match to the EACS at the weaker ORC binding site than ARS1 B2. **c**. Schematic figure of staged reaction, where ORC only remains bound to high affinity sites after stringent washing with 150 mM NaCl. **d**. ORC remains bound to the template DNA on both 90 bp and 90bp-B2 for up to 60 min in the presence of a 50X excess of competitor DNA. In lanes 1 and 9, competitor DNA was added together with ORC during preincubation. **e**. Orc2∆N loads MCM as efficiently as wtORC in a staged reaction. The data is plotted as mean ± SD of three technical replicates. Source data

The two key ORC binding sites at ARS1 (A/B1 and B2) are so close together that ORC cannot bind both sites simultaneously^6^. Therefore, MCM loading must occur in two sequential ORC binding steps. As shown in Fig. 2a, we arranged budding yeast origins according to the distance between the best match to the EACS (A domain) and the best predicted secondary ORC site in the opposite orientation (B2-like domain). In roughly one-third of yeast origins, the two ORC binding sites are as close or closer together than A/B1 and B2 in ARS1 (Fig. 2a). Using synthetic origins with two high-affinity ORC sites (perfect EACS/B1) in which distances are measured from the inside edges of the two B1 elements, 10 bp corresponds to the distance between A/B1 and B2 in ARS1, and this construct, like ARS1, can only bind a single ORC molecule (Extended Data Fig. 2a, lanes 5 and 6). Moving binding sites just 2 bp farther apart, however, allows two ORC molecules to bind simultaneously (Extended Data Fig. 2a, lanes 7 and 8). Thus, roughly two-thirds of yeast origins are predicted to allow simultaneous binding of two ORC molecules (Fig. 2a), unlike ARS1. In approximately 20% of yeast origins, the two predicted ORC binding sites are far enough apart to fit a single MCM hexamer between them, and in 23%, they are far enough apart to fit a DH between them. In only 15% of origins is the weaker ORC binding site a better EACS match than ARS1 B2 (Extended Data Fig. 2b). Therefore, asymmetry in ORC binding affinities appears to be a general property of yeast origins regardless of whether or not the two sites overlap. The MCM loading mechanism has primarily been studied using ARS1; it remains unclear whether the mechanism of MCM loading is the same at all origins or whether other mechanisms may be important at origins with different ORC binding affinities or wider spacing between ORC binding sites.

**Extended Data Fig. 3:**
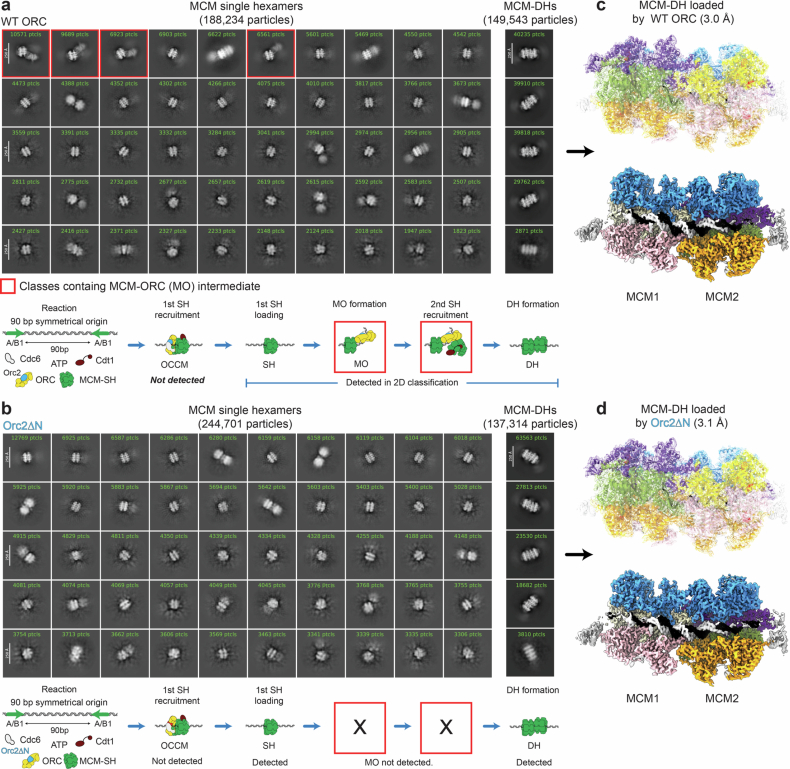
Orc2∆N cannot form MO intermediates on 90 bp origins containing symmetrical high-affinity ORC binding sites. 2D classification of cryo-EM particle images containing SHs and DHs loaded by wt (**a**) or Orc2∆N (**b**). Data were collected from early time-point (2 minute) MCM loading assays designed to enrich for MO intermediates. Green numbers indicate the particles contributing to each class average. Classes containing MO or MO-like particles were not detected in the experiment containing Orc2∆N. Cryo-EM density maps of DHs loaded by wt (**c**) or Orc2∆N (**d**). Top panels show EM maps with a semi-transparent surface colour zoned according to the DH atomic model described in Extended Data Fig. 5a and 10. Bottom panels show colour zoned EM maps with Mcm7,4,6 (MCM1) and Mcm2,5 (MCM2) removed to display DNA density in the DH central channel. DHs assembled on ARS1 or 90 bp symmetrical origins, with wt ORC, CDK phosphorylated ORC or Orc2∆N all appear identical. The displayed EM density maps have been density modified using EMReady.

To begin to address this question, we tested the ability of Orc2∆N, which cannot form MO at ARS1, to load MCM on a series of synthetic origins with two high-affinity ORC binding sites in the opposite orientation, placed at distances 10–300 bp apart. As shown previously^5^, WT ORC can load MCM efficiently on this series of origins, with a peak of MCM loading at 70–90 bp, but with efficient loading even at 300 bp. Orc2∆N cannot load MCM efficiently at ARS1, but surprisingly, loads MCM at all distances from 10–300 bp as efficiently as WT ORC (Fig. 2b). To determine whether Orc2∆N can promote MO formation in the context of origins with two high-affinity ORC binding sites, we performed cryo-EM on early time-point (2 min) MCM loading reactions with either WT ORC or ORC containing Orc2∆N using the 90 bp synthetic origin. Although MO particles were amongst the most abundant single hexamer (SH)-containing particles with WT ORC, they were not detected with Orc2∆N, even though similar numbers of DHs were detected (Fig. 2c and Extended Data Fig. 3), consistent with the biochemical data. Approximately 3 Å resolution structures of DHs, formed with WT ORC and Orc2∆N, appear identical (Extended Data Fig. 3). Moreover, DHs formed on the 90 bp origin with Orc2∆N are fully competent to support replication with purified proteins (Fig. 2d). Therefore, Orc2∆N cannot form MO, even on a template with two high-affinity sites, but can load functional DHs very efficiently. This shows that MO is not required when MCM is loaded from two high-affinity sites, regardless of whether those sites effectively overlap (10 bp) or are separated by a relatively large distance (300 bp). To test this concept directly, we changed one high-affinity EACS/B1 site in the most efficient synthetic template (90 bp) to the B2 element from ARS1, a lower-affinity ORC site (Fig. 2e). As shown in Fig. 2f, WT ORC loads MCM as efficiently on this template as it does on the parent template with two high-affinity sites, demonstrating that weak ORC sites can be effectively used even when the two ORC sites do not overlap. MCM loading with Orc2∆N on the 90 bp template was at least as efficient as WT, but MCM loading on the 90bp-B2 construct was greatly reduced. From these experiments, we conclude that the MO intermediate is critical for MCM loading when one of the ORC binding sites is of lower affinity, and, therefore, that the essential role of MO in DH assembly is to stabilize ORC binding at weak sites after first hexamer loading. Furthermore, MO is still essential when the high-affinity and low-affinity ORC sites are 90 bp apart, indicating that MO is not just required when ORC sites overlap, as in ARS1. However, MO is not required when MCM is loaded from two high-affinity ORC binding sites.

Given that the EACS/B1 sequences in both 90 bp and 90bp-B2 are high-affinity ORC binding sites, ORC binding to these sequences is stable under stringent (150 mM NaCl) washing, whereas binding to B2 is not^5^, which allows us to examine the efficiency of the ‘one ORC’ loading via ORC flipping versus the two ORC MO-independent mechanism in which a second ORC molecule loads the second hexamer. To test this concept, we pre-bound different concentrations of ORC to either the 90 bp or the 90bp-B2 origin; free ORC along with B2-bound ORC was then washed off (Extended Data Fig. 2c). Under these conditions, ORC remains stably bound to the high-affinity EACS/B1 element in both templates in the presence of excess competitor DNA for at least 60 min (Extended Data Fig. 2d). Finally, we added Cdc6 and MCM–Cdt1 and examined MCM loading. MCM loading under these conditions on the 90bp-B2 origin defines the maximal efficiency of MCM loading from a single ORC molecule. At saturating ORC concentrations, all binding sites in the 90 bp origin are bound, so there are no free binding sites for ORC to flip onto after first hexamer loading, and, therefore, all loading must occur via two ORC molecules. That this loading does not occur via the MO-dependent pathway is supported by the fact that Orc2∆N is as efficient as WT ORC at MCM loading from the two-site template under these conditions (Extended Data Fig. 2e). By contrast, loading on the 90bp-B2 template is strongly reduced with Orc2∆N (Fig. 2f) and, under the staged conditions, increases only linearly with increasing ORC concentration (Fig. 2g). As shown in Fig. 2g, there was approximately tenfold more loading on 90 bp than 90bp-B2 at high ORC concentrations. This result is consistent with the idea that a single ORC can load a DH from a single high-affinity site via ORC flipping and MO formation, but also shows that this pathway is less efficient than the pathway in which each MCM-SH is loaded by separate ORC molecules at two high-affinity sites. We note that at low ORC concentrations (<5 nM), loading on 90 bp and 90bp-B2 is similar. At these concentrations, loading on 90 bp is also predominantly via the one-ORC pathway, suggesting that B2 works as well as EACS/B1 as the second site in this pathway, consistent with the idea that MO stabilizes ORC binding to the weaker B2 site. These experiments imply that once the first hexamer is loaded, some subsequent step(s) in the one ORC mechanism—ORC release from A/B1, SH sliding away from B2, ORC rebinding B2 or second hexamer loading from the MO complex—have some inherent inefficiency. Taken together, we distinguish three separate ways MCM can be loaded: one ORC via MO, two ORC via MO and two ORC MO-independent.

### CDK phosphorylation of Orc2 blocks MO formation

**Fig. 3:**
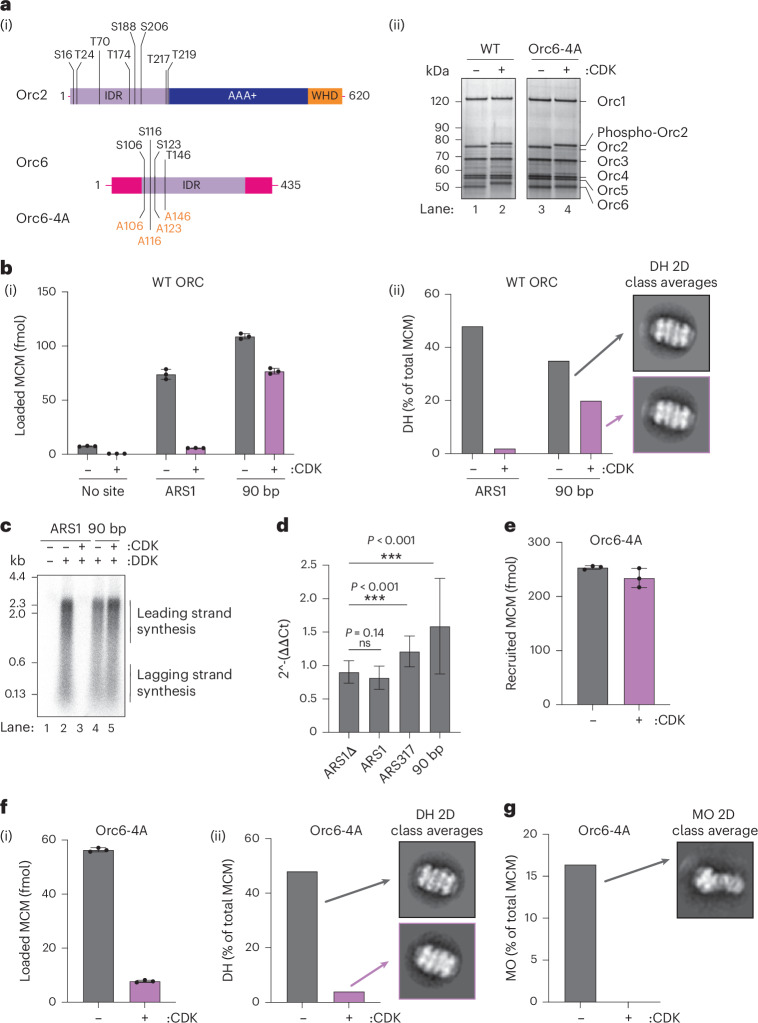
CDK phosphorylation of Orc2 inhibits the formation of MO. **a**, (i) Potential CDK phosphorylation sites (consensus sequences (^S^/_T_PX^K^/_R_)) in Orc2 and Orc6. (ii) An Orc6 mutant with four CDK target sites mutated to alanine (Orc6-4A) can be stably incorporated into the ORC complex but not phosphorylated. CDK phosphorylation of Orc2 in Orc6-4A-containing ORCs is comparable to WT. **b**, MCM loading on ARS1, but not 90 bp origins, is highly sensitive to CDK phosphorylation of ORC. The data in **b**(i) are plotted as the means of three technical replicates; error bars, s.d. Quantification of micrographs, total MCM and DH in negative-stain EM 2D classification data in **b**(ii): ARS1 − CDK (136, 3,531, 853); ARS1 + CDK (184, 4,748, 46); 90 bp − CDK (121, 3,392, 594); 90 bp + CDK (121, 4,587, 461). **c**, MCMs loaded onto 90 bp origins by phosphorylated ORC are replication competent. **d**, In yeast cells dependent on ORC phosphorylation to prevent re-replication, 90 bp spaced origins inserted into the yeast genome re-replicate, causing an increase in copy number, as detected by qPCR. The data are plotted as means of three biological replicates, with each containing two technical replicates of triplicates; error bars, s.d.; *n* = 18. *P* values were obtained from two-tailed *t*-tests (ns, not significant). **e**,**f**, ORC phosphorylated on Orc2 by CDK (Orc6-4A mutant) efficiently recruits MCMs to ARS1 origins (**e**) but is inhibited in MCM loading (**f**). The data in **e** and **f**(i) are plotted as means of three technical replicates; error bars, s.d. Quantification of micrographs, total MCM and DH in negative-stain EM 2D classification data in **f**(ii): ARS1, Orc6-4A − CDK (128, 3,044, 724); ARS1, Orc6-4A + CDK (174, 5,971, 126). **g**, ORC phosphorylated on Orc2 by CDK (Orc6-4A mutant) fails to form MO. Quantification of micrographs, total MCM and MO in negative-stain EM 2D classification data: ARS1, Orc6-4A − CDK (159, 4,482, 735); ARS1, Orc6-4A + CDK (266, 7,115, not detected). Source data

**Extended Data Fig. 4:**
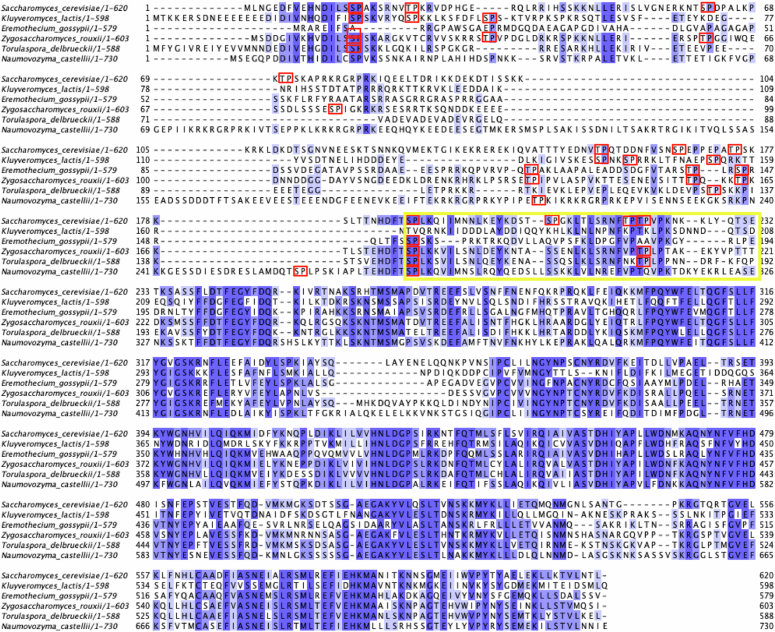
Closely related yeast species contain multiple potential CDK phosphorylation sites in the Orc2 IDR. The region of the Orc2 IDR that is predicted to form part of the MCM-ORC interface (yellow box) is moderately conserved and harbors multiple potential CDK phosphorylation sites (highlighted in red).

**Extended Data Fig. 5:**
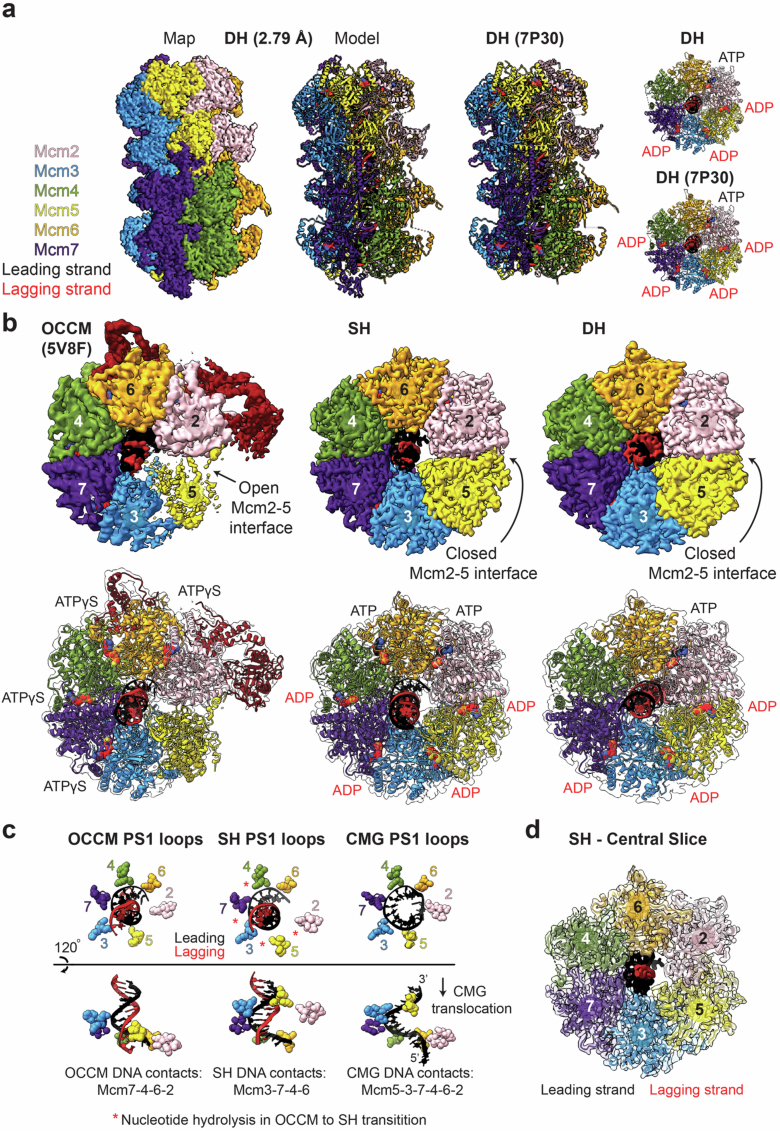
Comparison of DHs assembled by ORC and ORC phosphorylated on Orc2. **a**. Cryo-EM structure and atomic model for DHs assembled by ORC phosphorylated on Orc2 (left; this study) and atomic model for DHs assembled by unphosphorylated ORC (right; PDB: 7P30 ref. ^58^). The DH EM density map displayed has been density modified using EMReady^45^. **b**. Comparison of the MCM ring in the OCCM (left), SH (middle) and DH (right), showing that the SH and DH have fully closed Mcm2-5 interfaces and both appear to be in post-ATP hydrolysis states relative to the OCCM. **c**. Comparison of the PS1 loops of the OCCM (5V8F^46^**;** left), SH (this study; middle) and CMG (6SKO^66^; right), showing that the SH PS1 loops adopt a staircase configuration and engage the backbone of the leading strand DNA template like the CMG. **d**. Central slice through the SH structure, highlighting the well resolved EM density for the bases in the leading strand DNA template.

CDK prevents MCM loading outside of the G1 phase by promoting Cdc6 proteolysis^17–20^ and MCM–Cdt1 nuclear export^21–25^ as well as by inhibiting the ability of ORC to assemble DHs^26–28^. The region in the Orc2 IDR predicted to form part of the MCM–ORC interface in MO (Fig. 1c and yellow box in Extended Data Fig. 4) is moderately conserved among closely related budding yeasts, which all contain multiple potential CDK phosphorylation sites near the predicted interface with MCM in MO (Fig. 1c, red dots). Therefore, we reasoned that CDK phosphorylation of Orc2 might inhibit MCM loading by interfering with MO formation. Phosphorylation of WT ORC with S-phase CDK (Clb5–Cdc28–Cks1) caused a reduction in mobility of both the Orc2 and Orc6 subunits in SDS–PAGE (Fig. 3a); this CDK-phosphorylated ORC was very inefficient in DH formation at ARS1 (Fig. 3b), consistent with previous work^26–28^. Similar to Orc2∆N, however, phosphorylated ORC loaded MCM almost as well as unphosphorylated ORC on the 90 bp synthetic origin (Fig. 3b). To ensure that this loaded MCM was functional, we tested its ability to support DNA replication in vitro with purified proteins. Although pre-phosphorylated ORC did not support replication from an ARS1-containing template (compare lanes 2 and 3 in Fig. 3c), it supported replication from a template containing the 90 bp origin almost as well as unphosphorylated ORC (lanes 4 and 5, Fig. 3c), indicating that the DH formed is functional. Consistent with this idea, a 2.8 Å resolution cryo-EM structure of DH assembled with phosphorylated ORC is virtually identical to DH assembled with unphosphorylated ORC (Table 1 and Extended Data Fig. 5a). To test whether this 90 bp origin could also bypass CDK regulation of ORC in vivo, we reasoned that we might see re-replication from this origin in cells where Cdc6 and Cdt1–MCM regulation by CDK was bypassed, leaving cells to rely on CDK regulation of ORC to prevent re-replication^29^. Bypassing Cdc6 and Cdt1–MCM regulation is necessary because even in the MO-independent pathway, Cdc6 and Cdt1 are required for SH loading. We used a yeast strain in which CDK regulation of MCM was constitutively eliminated by fusing an unregulated nuclear localization sequence (SV40-TAg NLS) to Mcm7, and CDK regulation of Cdc6 was conditionally deregulated with a copy of a stable version of Cdc6 (Cdc6∆NT) under a galactose-inducible promoter. When both Cdc6 and MCM are deregulated, cells rely entirely on WT ORC phosphorylation to prevent re-replication^29^. We integrated a series of origins in place of ARS419, then arrested cells in G2/M, expressed stable Cdc6 and examined copy number after 3 h. As shown in Fig. 3d, deregulation of Cdc6 and MCM did not lead to any increase in DNA copy number of ARS1; ARS317, which had been previously shown to re-replicate under similar conditions^30^, showed elevated copy number, but the 90 bp origin showed the largest increase in copy number after Cdc6 deregulation, consistent with the idea that this synthetic origin bypasses CDK regulation of ORC both in vitro and in vivo.

**Table 1:** Cryo-EM data collection, refinement and validation statistics

	MCM-SH (EMD-19187) (PDB 8RIG)	MCM-DH (EMD-19186) (PDB 8RIF)
**Data collection and processing**		
Magnification	×130,000	×130,000
Voltage (kV)	300	300
Electron exposure (e^−^ Å^−2^)	51.4	51.4
Defocus range (mm)	1.0–5.0	1.0–5.0
Pixel size (Å)	1.08	1.08
Symmetry imposed	C1	C2
Initial particle images (no.)	621,909	178,188
Final particle images (no.)	396,366	135,143
Map resolution (Å)		
RELION (PostProcess)	3.41	2.8
FSC threshold	0.143	0.143
Resolve cryo-EM	3.14	2.60
FSC_ref_ threshold	0.5	0.5
Map resolution range (Å)	2.2–4.0	2.2–3.8
**Refinement**		
Initial model used (PDB code)	8RIF	7P30
Map resolution (Å)	2.6	3.0
FSC_ref_ threshold	0.5	0.5
Map sharpening *B* factor (Å^2^)	Resolve cryo-EM (27.3)	Resolve cryo-EM (0)
Model composition		
Non-hydrogen atoms	30,221	64,145
Protein residues	3,721	7,820
Nucleotides	38	106
Ligands (ATP, ADP, Mg^2+^, Zn^2+^)	2 ATP, 4 ADP, 6 Mg^2+^, 5 Zn^2+^	2 ATP, 6 ADP, 8 Mg^2+^, 10 Zn^2+^
*B* factors (Å^2^)		
Protein	65.37	61.85
Nucleotide	20.00	102.70
Ligand	57.35	63.70
R.m.s. deviations		
Bond lengths (Å^2^)	0.007	0.007
Bond angles (^o^)	1.117	1.232
**Validation**		
Molprobity score	1.26	1.30
Clashscore	2.04	1.81
Poor rotamers (%)	0.97	0.98
Ramachandran plot		
Favored (%)	95.88	94.87
Allowed (%)	3.96	4.98
Disallowed (%)	0.16	0.16

We next sought to determine which step in DH assembly was blocked by Orc2 phosphorylation. To examine the effects of Orc2 phosphorylation alone, we mutated four serine/threonine residues in the CDK consensus sequences (^S^/_T_PX^K^/_R_) in Orc6 to alanine to generate Orc6-4A. As shown in Fig. 3a, the phosphorylation-dependent shift of Orc6 was greatly reduced in Orc6-4A, but Orc2 was still shifted by CDK phosphorylation. On ARS1, CDK phosphorylation of Orc2 did not affect MCM recruitment in ATPγS (Fig. 3e). However, Orc2 phosphorylation led to a substantial reduction in DH formation in both DNA-pulldown (Fig. 3f(i)) and EM-based (Fig. 3f(ii)) assays and a complete inhibition of MO formation (Fig. 3g), indicating that phosphorylation of Orc2 alone is sufficient to inhibit DH formation. Thus, CDK phosphorylation of Orc2, like deletion of the Orc2 IDR (Fig. 1), inhibits DH assembly before MO formation, consistent with recent work^13^. Although CDK phosphorylation of Orc2 blocks MO formation and DH formation on the ARS1 origin, it does not block DH formation on the 90 bp origin (Fig. 3b), consistent with the results in the previous section showing that MO is not required when origins contain two high-affinity binding sites (Fig. 2b,f).

### Structure of the MCM-SH

**Fig. 4:**
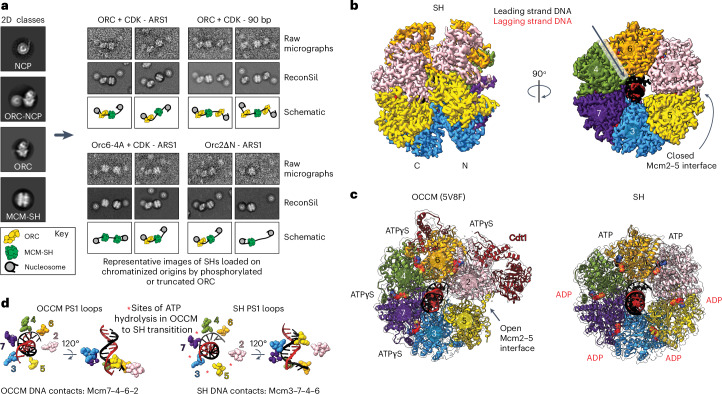
Structure of the MCM-SH. **a**. Negative-stain EM ReconSil of MCM loading reactions performed with phosphorylated or truncated Orc2. Left, conventional EM 2D class averages. Right, representative images of chromatinized origins bound by single MCM helicases with or without DNA-bound ORCs and flanked by nucleosomes, shown as raw micrographs (top rows), after ReconSil (middle rows) or as schematics (bottom rows). **b**, Cryo-EM structure of a DNA-bound SH. **c**, Cryo-EM structures and atomic models for OCCM (PDB 5V8F (ref. ^46^)) and a SH. The EM density map displayed for the SH has been density-modified using EMReady^45^. **d**, ATP hydrolysis in the Mcm4–7, Mcm7–3, Mcm3–5 and Mcm5–2 ATPase sites during SH loading is associated with a reconfiguration of the MCM pre-sensor 1 (PS1) loops into a staircase configuration that engages the leading-strand template DNA backbone.

Formation of MO requires Orc2 and Orc6 to bridge the N-terminal interface between the Mcm2 and Mcm5 subunits that form MCM’s DNA entry ‘gate’. However, it is currently unknown whether closure of the Mcm2–5 interface precedes ORC binding in the MO or whether ORC binding closes the Mcm2–5 gate to load the first MCM hexamer. Phosphorylated Orc2 (Orc6-4A) recruits MCM normally but does not form MO, suggesting that Orc2 phosphorylation either blocks a step in the loading of the SH (for example, OCCM disassembly) and/or directly prevents ORC binding to a loaded SH during the formation of MO. To investigate, we performed single-particle reconstitution in silico (ReconSil)^11^ experiments to visualize entire chromatinized origins of replication during MCM loading reactions containing phosphorylated or truncated Orc2. ReconSil experiments enabled us to identify SHs that were apparently trapped on DNA between nucleosomes (Fig. 4a). Thus, the Orc2 IDR seems not to be required for OCCM assembly or disassembly to release SH onto DNA but is required for MO formation, which can be inhibited by CDK phosphorylation of the Orc2 IDR.

To confirm this idea, we used cryo-EM to visualize the helicase loading reaction with CDK-phosphorylated Orc2. The 3.4 Å resolution structure (3.1 Å after density modification) shows a single MCM ring encircling duplex DNA (Fig. 4b, Table 1 and Extended Data Fig. 5). Unlike the MCM in the OCCM structure, the SH contains a completely closed Mcm2–5 interface identical to that seen in the DH (Fig. 4b and Extended Data Fig. 5b). Thus, phosphorylation of ORC2 blocks DH formation after a SH has been fully closed around DNA, implying that ring closure precedes and is not a consequence of the N-terminal ORC binding that results in MO formation. Comparison with MCM in the OCCM complex reveals that the DNA grip in the helicase ring changes after closure of the Mcm2–5 gate (from Mcm7–4–6–2 to Mcm3–7–4–6 duplex engagement), probably as a consequence of ATP hydrolysis (Fig. 4b). Nucleotide occupancy changes from ATPγS in the 3–7, 7–4, 4–6 and 6–2 interfaces in OCCM, to ATP in the 4–6 and 6–2 interfaces and ADP in the 2–5, 5–3, 3–7 and 7–4 interfaces in SH (Fig. 4c). This change in nucleotide occupancy indicates a minimum of four ATP hydrolysis events that may drive the re-orientation of the ATPase pore loops, with pre-sensor 1 loops arranged in a staircase configuration that follows the helical rise of the leading-strand template (Fig. 4d), as observed in the active CMG helicase (Extended Data Fig. 5c). Despite leading-strand DNA engagement by the pre-sensor 1 loops, our data do not indicate that SH actively translocate on DNA; instead, our ReconSil data (Fig. 4a) show SH randomly distributed across origins, having moved either in an N-terminal or C-terminal direction after loading. This result is consistent with single-molecule studies showing SH diffusion following loading by ORC^31,32^.

### Mechanism of MO-independent DH assembly

**Fig. 5:**
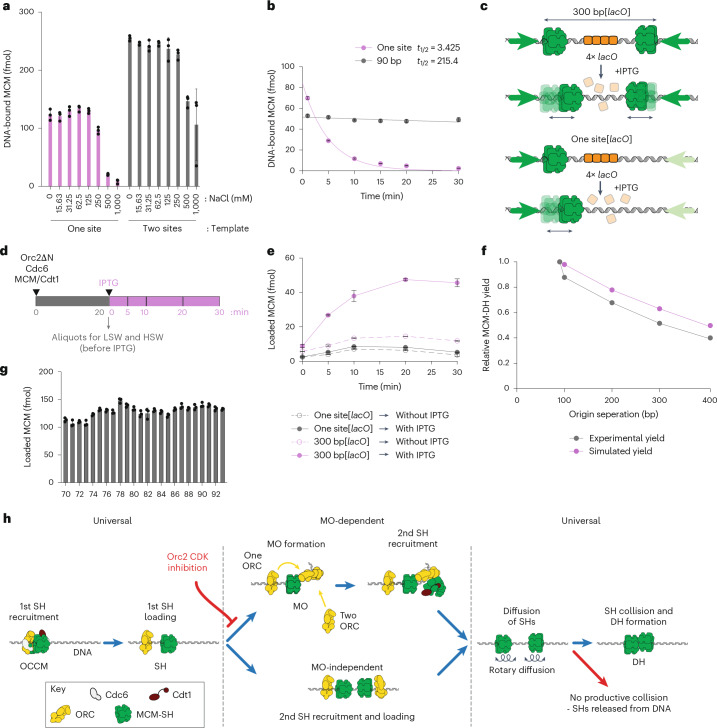
Mechanism of MO-independent DH formation. **a**,**b**, SHs have a lower salt stability (**a**) and a shorter half-life (*t*_1/2_) (**b**) on DNA than DHs. The data in **a** and **b** are plotted as means of three technical replicates; error bars, s.d. **c**,**d**, Schematics illustrating origins (**c**) and assay (**d**) used for characterizing MO-independent DH formation via collision of two independently loaded SHs. **e**, Aliquots were taken at different time points for HSW. The amount of MCM was significantly increased on the 300 bp[*lacO*] template after HSW, indicating that two independently loaded SHs formed a DH. In the presence of IPTG, approximately 3.8-fold more MCM was detected on the 300 bp[*lacO*] at 30 min compared to the reaction without IPTG. The data are plotted as means of three technical replicates; error bars, s.d. **f**, Efficiency of DH formation decreases with increase in separation between ORC binding sites and can be modeled as the meeting probability of two diffusing SHs loaded at ORC binding sites at a given separation in the experimental time (20 min). The experimental yield data were obtained from the mean of three datasets. **g**, MCM loading is insensitive to the separation distance between two ORC binding sites (between 70 and 93 bp) and relative orientation of recruited ORCs with respect to each other along the DNA helix. The data are plotted as means of three technical replicates; error bars, s.d. **h**, Proposed model for MCM loading via MO-dependent and independent pathways. Both pathways converge on a ‘universal’ mechanism that requires loading of two SHs that must collide in a head-to-head orientation to form a DH. Source data

To examine the properties of the loaded SH, we assembled MCM with Orc2∆N on either a one-site template, in which only SH assembles, or a template containing two ORC binding sites separated by 90 bp, in which DH efficiently forms. We then challenged the loaded MCM products with buffers containing different salt concentrations. As shown in Fig. 5a, MCM loaded on both templates was resistant to salt concentrations up to 250 mM NaCl; virtually all of the MCM was removed from the one-site templates at 500 and 1,000 mM NaCl, whereas about 40% of the MCM remained on the 90 bp template after 500 and 1,000 mM NaCl washes. These experiments indicate that SH is stable up to ~250 mM NaCl but is efficiently removed at salt concentrations of 500 mM NaCl and above. The DH is stable up to 2 M NaCl^33^, so the fraction of MCM removed by 500 mM NaCl from the 90 bp origin probably represents SH, with the MCM remaining at 500 and 1,000 mM NaCl being DH. This suggests that a substantial fraction of the MCM loaded even on this very efficient origin are SHs that have not matured into DHs. This is consistent with single-molecule experiments showing that SHs are readily detected during normal MCM loading reactions^31^.

To determine the off rate of SH under MCM loading conditions (low salt), MCM was loaded as above using Orc2∆N on a single-site sequence, and excess protein was removed by low-salt wash (LSW). The amount of SH remaining on DNA was then assessed after incubation for the indicated times, followed by another LSW. Figure 5b shows that SHs were lost from DNA with a half-life of approximately 3.5 min. To compare this to the DH, we loaded MCM onto the 90 bp template and then washed free proteins and SHs off with high salt (1 M NaCl) before taking time points. As shown in Fig. 5b, DHs are stable for the entire duration of the experiment. Therefore, the SH is much less stable than the DH on DNA.

**Extended Data Fig. 6:**
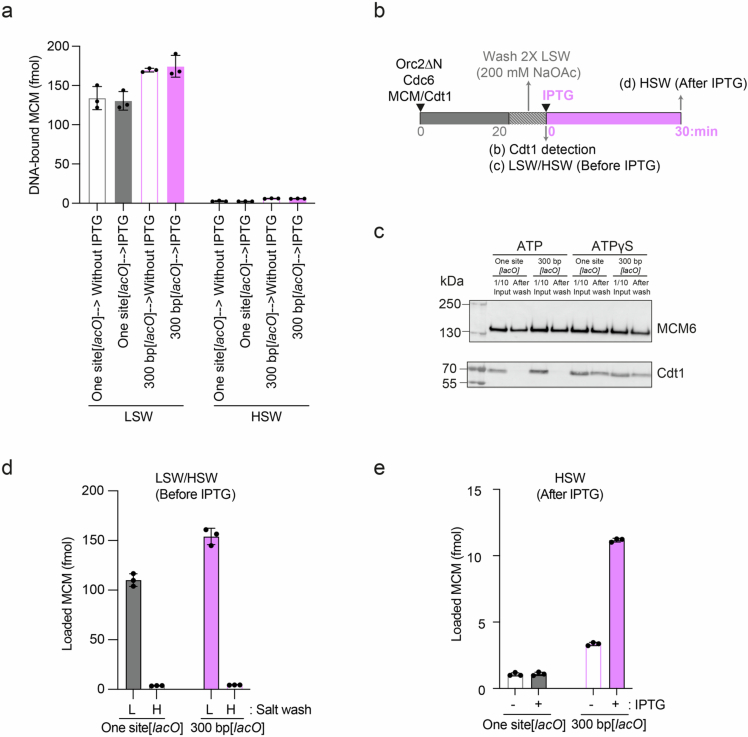
DH formation by two single hexamers independent on free MCM-Cdt1. **a**. MCM loading assay performed with 20 min incubation of Orc2∆N, Cdc6 and MCM-Cdt1 with ATP and a DNA template containing two high affinity ORC sites separated by 300 bp and 4 lac operators. Aliquots taken before IPTG addition in Fig. 5d show MCM loaded onto DNA (detectable after LSW), but no HSW stable DH formation. The data is plotted as mean ± SD of three technical replicates. **b**. Schematics illustrating the assay used for MO-independent DHs formation in panel c-e. Orc2∆N, Cdc6, MCM and Cdt1 were incubated for 20 min in the presence of LacI, then two washes with LSW (200 mM NaOAc) were introduced before the addition of IPTG to wash off free MCM-Cdt1 and Cdc6. **c**. Aliquots were taken before IPTG addition for immunoblot detecting Cdt1, indicating that most of free MCM-Cdt1 in the solution was washed off. Mcm6 is used as a control. **d**. Aliquots were taken after extensive washes but before IPTG addition for both LSW and HSW, showing that MCM was loaded onto DNA (LSW) without forming DH (HSW). The data is plotted as mean ± SD of three technical replicates. **e**. After incubation with IPTG, the samples were washed with HSW to detect DH formation. In the presence of IPTG, approximately 3.3-fold more MCM was detected on the 300 bp[*lacO*] compared to the reaction without IPTG. The data is plotted as mean ± SD of three technical replicates. Source data

To show more directly that two independently loaded SHs can assemble into a DH, we generated a template with two high-affinity ORC sites separated by 300 bp and four lac operators between the sites (Fig. 5c). As a control, we also generated a template with a single ORC site and four lac operators. We pre-assembled SHs on these templates with Orc2∆N for 20 min (Fig. 5d). As shown in Extended Data Fig. 6a, there is considerable MCM loaded on both templates at this point; however, this MCM is all removed by high-salt wash (HSW), indicating that there are no DHs. DH formation (high-salt-resistant MCM) was detected at significant levels only on the two ORC binding site templates and only after IPTG addition (Fig. 5e) in a time-dependent manner. This strongly supports the idea that two SHs can generate a DH after interaction. To exclude the possibility that loading occurs via MO, we repeated the experiment in Fig. 5e except we removed all free MCM–Cdt1 and Cdc6 by washing the beads before adding IPTG (Extended Data Fig. 6b). Extended Data Fig. 6c shows that these washes effectively remove all Cdt1 from reactions before IPTG addition in reactions containing ATP, but not ATPγS, where Cdt1 remains as part of the OCCM complex. Extended Data Fig. 6d shows the amount of MCM loaded onto DNA in ATP (one-site and two-site) after salt washes and shows that all of this MCM is removed by HSW, consistent with the complete lack of DH. Extended Data Fig. 6e shows high-salt-resistant MCM loading after IPTG addition, confirming that DH assembly occurs entirely from SHs that were loaded before IPTG addition without a requirement for Cdc6, Cdt1 or free MCM. The overall amount of DH assembled in this experiment (~12 fmol) is lower than the amount formed in Fig. 5e (~45 fmol). We believe that this is a consequence of the loss of SHs during washing before IPTG addition. The presence of 150 fmol of SH after washes at the point of IPTG addition (Extended Data Fig. 6d), distributed randomly on 300 fmol ORC binding site, predicts that only 25% of DNA molecules (37.5 fmol) at this point have two SHs, a prerequisite for DH assembly. Furthermore, because the two ORC sites in this template are 300 bp apart, we would expect a further reduction in loading efficiency of around 50% (see below). Consequently, the maximum loading we might expect under these conditions should be less than 20 fmol. Taken together, these experiments show that DH can assemble from loaded SHs in the absence of Cdc6, Cdt1 and free MCM.

**Extended Data Fig. 7:**
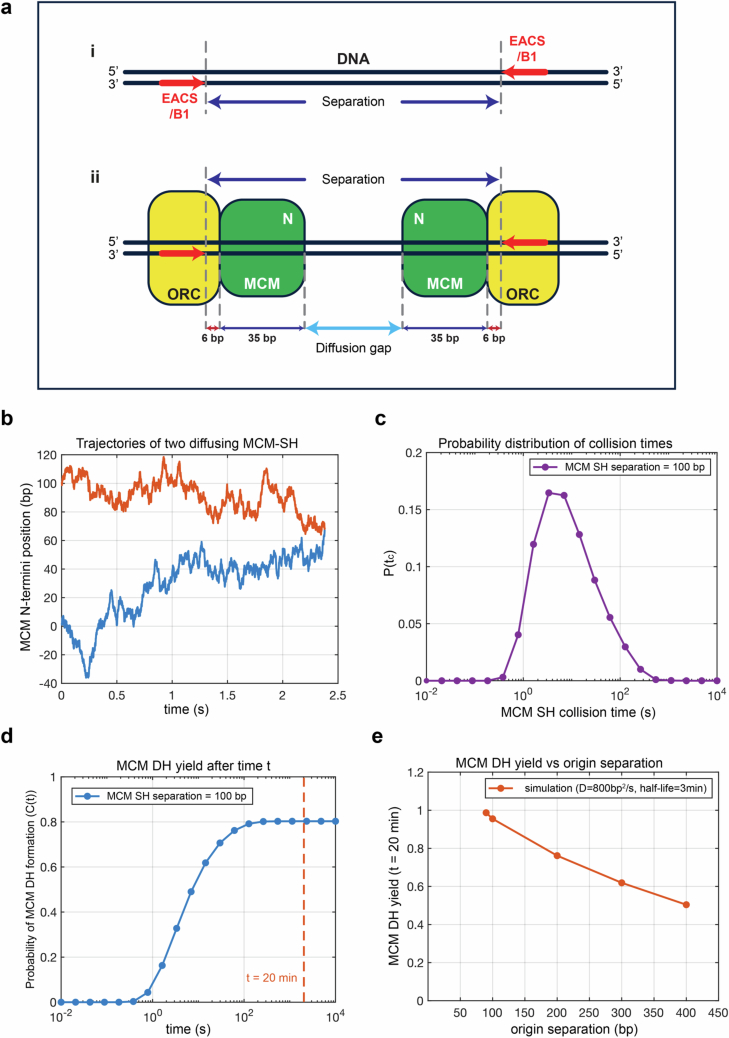
Simulating efficiency of MCM DH formation. **a**. Schematic of the simulated system and definition of relevant distances. i.The separation between two origins oriented towards each other on the DNA is defined as the distance in base pairs between the 5’ ends of the origin sequences. ii. ORC binds to the origin sequence with an overhang of 6 base pairs in front of the 5’ end of the origin. MCM-SH binds in front of the ORC and occupies 35 base pairs with the distance between eh origin and the N-termini of MCM-SH being 6 + 35 = 41 base pairs. The distance between the N-termini of MCM-SHs is the diffusion gap that must be overcome to result in MCM-DH formation and is determined by the separation between origin sequences and the space occupied by ORC and MCM2-7 complexes on the DNA. **b**. an example of a simulated traces showing two MCM-SHs loaded at a separation of 100 base pairs which then diffuse along the DNA substrate and ultimately collide with each other resulting in MCM-DH formation. **c**. Probability distribution of MCM-SH collision times. **d**. Probability of MCM-DH formation with respect to time which is the. Cumulative sum of the MCM-SH collision time distribution shown in (**c**). MCM-DH yield is defined as the MCM-DH formation probability at a cutoff time determined by the incubation time in vitro experiments (in this case t_cutoff = 20 min). **e**. Simulated MCM-DH yield at various values of separation between origin sequences.

The efficiency of DH assembly decreases as the two ORC sites are moved farther than ~90 bp apart. We reasoned that the farther apart two SHs are loaded, the higher the probability that one or the other will fall off before meeting. To model this process, we used the observed one-dimensional diffusion constant for the DH^34^, together with the off rate determined in Fig. 5b (Extended Data Fig. 7), and, as shown in Fig. 5f, the predicted loading efficiencies are remarkably close to those seen in the experimental data. Diffusion could occur with rotation following the path of the DNA helix, or by diffusion without rotation. For several reasons, we favor the idea that movement occurs coupled with rotation tracking the DNA helix. First, the structure of the SH shows a single mode of DNA engagement within the MCM central channel, with individual nucleotides clearly resolved (Extended Data Fig. 5d). Despite the large number of particles (~400,000), further 3D classification failed to detect SHs with an alternate DNA engagement, suggesting that the DNA register remains the same in all imaged SHs, regardless of their relative position on the origin DNA. If SHs diffused without rotation, they would be unlikely to have a highly preferred mode of DNA engagement. Second, without rotation, two SHs would meet in different registers depending on how far apart the loading sites were. One might expect to see evidence of this in loading efficiency; however, Fig. 5g shows that loading efficiency is very similar at every distance from 70–93 bp. Rotation along the DNA helix ensures that the two SHs always meet with the same register; using the first steric clash between hexamers as the end point of rotational diffusion along B-form DNA, this register is very close to the register seen in the DH (Supplementary Video 1). Taken together, we propose that DH assembly occurs by random rotational diffusion and meeting of two SH along the DNA, followed by a presently uncharacterized ‘locking’ of the two into a stable DH.

## Discussion

Our results, summarized in Fig. 5h, indicate that MCM can be loaded by at least three distinct pathways in budding yeast. In each pathway, the unifying mechanism is the loading of two SHs with inverted orientations. DH assembly can be coordinated by the MO, which facilitates ORC recruitment to a weak DNA site. Alternatively, DH assembly can be done in the absence of the MO, in which case two independently loaded SHs translocate by rotation along the helical axis until meeting to form a stable DH. The loading of the SH defines a precise register of DNA engagement that ensures two loaded SHs with inverted orientations will always meet in the same subunit register, regardless of the distance between their loading sites. Our data (Fig. 2b) show that DH formation is at least as efficient without MO as with MO, indicating that the SH to DH ‘locking’ mechanism is intrinsic to two correctly oriented and DNA-engaged SHs and is independent of the MO. We cannot rule out the possibility that some SH movement is directional and coupled to ATP hydrolysis; however, for two reasons, we favor a mechanism whereby DH formation occurs upon collision of two SHs that randomly slide along the helical axis of DNA. First, because of the wide distribution in distances between ORC binding sites at origins, it is sometimes necessary for SH to move in a C-terminal direction (for example, at ARS1) and sometimes in an N-terminal direction. A priori, this is best accommodated by random motion. Second, our ReconSil images show SH that have been imaged after movement in either direction from their loading site (Fig. 4a). If SH moved directionally, we would expect to see them accumulate with their N-termini adjacent to nucleosomes (or a downstream ORC). Instead, we find SH at a range of positions, including instances in which the SH has moved in a C-terminal direction to occupy the site previously occupied by ORC. This result is consistent with previous single-molecule studies showing MCM single hexamer diffusion after loading^31,32^.

SHs are considerably less stable on DNA than DHs. We suggest that this is because the Mcm2–5 interaction is intrinsically weak, providing a gate for DNA to exit the central channel. This could explain recent FRET experiments showing that the Mcm2–5 gate is not stably closed after first hexamer loading by ORC after Orc2 phosphorylation^13^. Although this was interpreted as meaning phosphorylation prevented ring closure, our results indicate that gate closure occurs, but the SH ring is unstable. The Mcm2–5 gates are not aligned in the DH but are located approximately 180° apart; therefore, even if both gates open at the same time, the DH would remain topologically bound around DNA^35^, which may prevent dissociation of the DH. Alternatively, contacts between the hexamers may stabilize the Mcm2–5 gate in the DH, preventing opening^13^. Our experiments have not addressed the role of Orc6 phosphorylation, which will be interesting to explore.

Previous work in yeast has shown that a substantial fraction of MCM is released from G1 chromatin with a moderate salt concentration (350 mM NaCl)^36^, suggesting the presence of SH on chromatin. From yeast to humans, the total amount of MCM bound to chromatin far exceeds the number of active replication origins. This ‘MCM paradox’ has been explained, at least in part, by the existence of dormant origins that are activated under stress conditions^37–40^; our results suggest that in addition, some of the excess MCM may be in the form of SH, which cannot support replication (for example, Figs. 2d and 3c). SHs are relatively short-lived, but there may also be active mechanisms to remove them from chromatin. Further work is required to assess what fraction of chromatin-bound MCM is SH.

Synthetic origins comprising two high-affinity ORC binding sites load MCM even more efficiently than ARS1 (for example, Fig. 2b) and function as autonomously replicating sequences in vivo^5^. Moreover, replication origins in *Archaea*, the progenitor replication system of eukaryotes, generally comprise multiple, high-affinity ORC sites in opposite orientations and at distances similar to our synthetic origins. However, yeast origins generally have a single high-affinity site flanked by low-affinity sites, indicating that MO-dependent DH assembly is likely to be the predominant mode of MCM loading in vivo. Our work suggests an explanation for this: origins with two high-affinity sites cannot be inhibited by CDK phosphorylation of Orc2 (Fig. 3c,d). We propose that this arrangement has been selected against during evolution: co-evolution of sequence-specific DNA binding by ORC^41,42^ and CDK regulation of ORC function has resulted in the asymmetry in modern yeast replication origins. Whether MO-dependent DH assembly in vivo proceeds via one or two ORC pathways is unclear, given that we do not know the concentration of free ORC in cells and currently lack a mutant that can distinguish these pathways. The ability of Orc2∆N or CDK-phosphorylated Orc2 to load low levels of DHs at endogenous origins like ARS1 may help explain why three other CDK targets (Orc6, Cdc6 and Mcm3) are required to completely block MCM loading outside of G1 phase in yeast.

Human Orc2 also contains a long N-terminal IDR^43^ but has no detectable sequence similarity to the yeast IDR. Recent work has shown that human ORC can load MCM through an MO-independent mechanism that does not require Orc6 and a mechanism that involves an MO-like intermediate. This mechanism does not appear to involve the Orc2 IDR, consistent with the hypothesis that CDK regulation of the MO by the Orc2 IDR co-evolved with the structure of yeast origins. Orc2 from multicellular plants entirely lacks an N-terminal IDR; therefore, plants are unlikely to use the same MO-dependent mechanism. Whether plants and metazoans use a mechanism related to the two ORC MO-independent mechanism or some other, novel mechanism requires further work. Finally, the recent sequencing of several metamonad genomes has shown that although all six MCM genes are present, organisms in the genus *Carpediemonas* lack all ORC subunits, Cdc6 and Cdt1 (ref. ^44^), indicating additional MCM loading mechanisms remain to be discovered.

## Methods

### Strains

All primers, plasmids and strains used for strain construction are listed in Supplementary Tables 1, 2 and 3, respectively. The YJL3239 strain was a gift from J. Li^29^. The ARS317, ARS1, ARS1Δ and synthetic 90 bp origins were inserted in ARS419 using a previously described method^30^ with some modifications. The endogenous ARS317 was deleted and replaced with kanMX6 from pFA6a-3HA-kanMX6 with amplification using CTL61 and CTL62 (yCTL5). URA3 was replaced with hphNT1 from pFA6a-hphNT1 with amplification using CTL98 and CTL99 (yCTL18). URA3 was amplified using pRS306 with primers CTL86 and CTL87 and additionally inserted in ARS419 at position 567 kb in Chromosome IV (yCTL19). The ARS317, ARS1, ARS1Δ and synthetic 90 bp origins were amplified from plasmid ARS317-Abf1(pCTL69), ARS1-Abf1 (pCTL68), ARS1Δ (pGC404 in ref. ^5^) and 90bp-Abf1 (pCTL67), respectively, using CTL160 and CTL161 and counter-selected with 5-FOA (Melford), resulting in strains yCTL30, yCTL29, yCTL28 and yCTL33. A total of 1 mg ml^−1^ of 5-FOA was added to minimal media containing 1× YNB, 2% glucose, 1× complete supplement mixture and 80 μg ml^−1^ uracil. The ARS317-RIP sequence was identified according to the L4-L12 region in a previous work^30^.

The sequence for the Orc6 phosphorylation mutant Orc6-4A was obtained from Invitrogen GeneArt Synthesis (Thermo Fisher Scientific). This sequence was then used to replace the WT Orc6 in the plasmid pJF18, as in a previous publication^27^, to create strain yCTL6 (Orc6-4A). Endogenous Orc6 was Flag-tagged to allow removal of the endogenous WT protein.

Amino acids 2–235 in Orc2 were deleted in the Orc2 N-terminal truncation mutant (Orc2ΔN). This mutant was introduced into pJF19, as in a previous publication^27^, to replace the WT Orc2. The WT Orc2 was Flag-tagged so that the endogenous protein could be removed. The resulting strain was named yCTL17.

### Protein expression and purification

In this study, ORC, Cdc6, Luc-MCM/Cdt1, CDK and Sic1 were expressed and purified according to previously described methods^5,26,27^. The Orc2∆N mutant was purified identically to WT ORC, but with the additional step of using Flag beads after CBP pulldown to remove endogenous WT proteins. The same was done for the Orc6 phosphorylation mutants.

### ORC binding assay

ORC binding assays were performed as previously described^5^. In brief, 0.25 pmol DNA of 0.6 kb PCR substrates with a single biotin (GC117 and GC298) were coupled with 5 μl of Dynabeads M-280 streptavidin (Invitrogen, 11205D) in 10 μl of binding buffer (5 mM Tris-HCl pH 8.0, 0.5 mM EDTA, 1 M NaCl), for 30 min at 30 °C with mixing at 1,250 rpm in a 1.5 ml microcentrifuge tube. Beads were washed twice and resuspended in buffer containing 10 mM HEPES-KOH pH 7.6 and 1 mM EDTA. The bead-bound DNA substrate was then incubated with 20 nM ORC in 40 μl loading buffer (25 mM HEPES-KOH pH 7.6, 100 mM NaOAc, 10 mM MgOAc, 0.02% NP-40, 5% glycerol, 1 mM dithiothreitol (DTT), 5 mM ATP, 80 mM KCl). The reaction was incubated for 15 min at 30 °C with mixing at 1,600 rpm on a 96-well plate. Reactions were then washed twice with LSW additionally containing 80 mM NaCl (45 mM HEPES-KOH pH 7.6, 5 mM MgOAc, 300 mM NaOAc, 0.02% NP-40, 10% glycerol, 2 mM CaCl_2_, 80 mM NaCl) for 2 min, 30 °C at 1,600 rpm. A total of 150 mM of NaCl was used in the washing step in Extended Data Fig. 2d. The washed beads were then washed once with LSW again before resuspending in 15 μl LSW with 600 units of micrococcal nuclease (MNase; NEB, M0247S) and incubated for 2 min, 30 °C at 1,600 rpm. The supernatant from the two reactions was collected and separated by Criterion XT Tris Acetate 3–8% (Bio-Rad) SDS–PAGE and visualized by silver staining.

### MCM loading

The MCM loading assay was performed as previously described^5^. Biotinylated DNA substrate (0.15 pmol) was attached to magnetic beads and then incubated with 12 nM ORC, 20 nM Cdc6, 80 nM Luc-MCM/Cdt1 in 40 μl loading buffer for 20 min at 30 °C with mixing at 1,600 rpm. Beads were washed twice with HSW (45 mM HEPES-KOH pH 7.6, 5 mM MgOAc, 0.02% NP-40, 10% glycerol, 1 M NaCl) for 2 min and twice with LSW (45 mM HEPES-KOH pH 7.6, 5 mM MgOAc, 300 mM NaOAc, 0.02% NP-40, 10% glycerol, 2 mM CaCl_2_) for 20 s. Proteins were released by 30 μl of LSW with MNase treatment for 2 min and transferred to white, flat-bottom, half-area 96-well plates (Corning, 3642). Nano-Glo Luciferase assay substrate (Promega N1120) was diluted 1:50, and 30 μl of diluted substrate was added to the sample. A standard curve was also prepared with a 2.5-fold dilution of Luc-MCM (ranging from 0.027–256.33 fmol). The luminescence was measured on a PHERAstar plate reader using ‘LUM Plus’ optic module (BMG Labtech) with the read time of 1 s per well at 12.1 mm focal height. The background (LSW only) was subtracted, and the amount of Luc-MCM was calculated using a linear regression of the standard curve of Luc-MCM. All experiments were done in triplicate. In the staged reaction depicted in Fig. 2g, ORC at various concentrations was preincubated with DNA beads for 15 min at 30 °C with mixing at 1,600 rpm. The reaction was washed twice with LSW + 150 mM NaCl buffer described above. Then, 40 nM Cdc6 and 80 nM MCM–Cdt1 were added to the reaction and incubated for another 5 min.

In the inducible roadblock assay, beads were resuspended in 20 μl binding buffer (25 mM HEPES-KOH pH 7.6, 100 mM NaOAc, 10 mM MgOAc, 0.02% NP-40, 5% glycerol, 5 mM DTT, 80 mM KCl). Then, 2.4 pmol of LacI (a gift from G. Cameron and H. Yardimci^47^) was added and incubated for 30 min at 30 °C with mixing at 1,250 rpm. The beads were washed twice and resuspended in 5 μl of binding buffer. This mixture was subsequently added to the MCM loading reaction and incubated for 20 min. Following this, 10 mM of IPTG and a 50× molar excess of competitor DNA containing one ORC binding site (EACS.B1 F and EACS.B1 R) were added. The reaction was aliquoted at the indicated time points for HSW, followed by LSW, as described above.

The conditions depicted in Extended Data Fig. 7 were the same as in Fig. 5e, except 9.6 pmol of LacI was used. After incubation with MCM for 20 min (before the addition of IPTG), beads were washed twice in 45 mM HEPES-KOH pH 7.6, 5 mM MgOAc, 200 mM NaOAc, 0.02% NP-40, 10% glycerol and 2 mM CaCl_2_. For the western blot, the samples were run on a 3–8% polyacrylamide gel (Invitrogen), immunoblotted and detected using Pierce ECL (Thermo Scientific) reagents. Polyclonal antibody JDI 70 was used to detect Cdt1 (ref. ^48^) at a dilution of 1:2,500. Polyclonal antibody against Mcm6 (E78) was raised against a peptide containing Mcm6 residues 1,005–1,017 and was used at a dilution of 1:1,000 in this assay.

### MCM recruitment

MCM recruitment assay was performed identically to the MCM loading assay, with the exception that 5 mM ATP was replaced with 5 mM ATPγS. After the incubation, the beads were washed twice with an ice-cold LSW for 20 s.

All experiments in the luciferase assays were done in triplicate, and the data are plotted as mean ± s.d.

### CDK phosphorylation of ORC

For CDK-phosphorylated ORC, 12 nM of ORC was incubated with 12 nM CDK for 10 min at 30 °C in loading buffer containing 0.5 mM ATP. To stop the reaction, 60 nM Sic1 was added and incubated for an additional 5 min at 30 °C. For non-phosphorylated ORC, 12 nM ORC was first incubated with 60 nM Sic1 for 5 min at 30 °C and then with 12 nM CDK for 10 min at 30 °C. The subsequent loading assay was done as described above. For Fig. 3a, proteins were separated by Criterion XT Tris Acetate 3–8% (Bio-Rad) SDS–PAGE and visualized by silver staining.

### Salt sensitivity assay

The salt sensitivity assay was performed identically to the MCM loading assay. After 20 min incubation, the samples were washed twice with cold LSW containing the indicated NaCl concentration (as shown in Fig. 5a) for 30 s, followed by two washes with cold LSW.

### Retention assay

The retention assay was performed identically to the MCM loading assay. After 20 min of incubation, the beads were washed with HSW (for 90 bp origin only) and then cold loading buffer (for both the one-site and 90 bp origins) for 40 s at 30 °C with mixing at 1,600 rpm. Then, 50× molar excess of competitor DNA in loading buffer (preheated at 30 °C) was added, and the mixture was aliquoted for an additional wash with HSW (for 90 bp origin only), followed by cold LSW (for both the one-site and 90 bp origins) at the indicated times.

### Replication assay

The replication assays were performed according to a previously described protocol^26^ with modifications. In brief, 48 nM ORC /Orc2∆N /phosphorylated ORC (pre-phosphorylation of Orc6-4A with 50 nM CDK for 10 min at 30 °C), 80 nM Cdc6, 320 nM MCM–Cdt1 and 16 nM DNA were incubated for 20 min at 30 °C, 1,250 rpm in reaction buffer containing 25 mM HEPES-KOH pH 7.6, 10 mM MgOAc, 2 mM DTT, 0.02% NP-40, 100 mM KGlu, 5 mM ATP and 80 mM KCl. Next, 50 nM DDK and 50 nM CDK were added with further incubation for 10 min. The mixture was diluted 4× in replication mix as follows: 20 nM Cdc45, 15 nM Dpb11, 20 nM Pol ε, 10 nM GINS, 50 nM RPA, 5 nM TopoI, 30 nM CDK, 50 nM Pol α, 13 nM Sld3/7, 10 nM Mcm10, 3 nM Sld2, 33 μM ^32^P-dCTP, 200 μM NTPs and 80 μM dNTPs for 30 min, 30 °C, 1,250 rpm. The reactions were then stopped by adding 50 mM EDTA (final concentration) and filtered through an Illustra Microspin G-50 column. The replication products were resuspended in loading buffer and separated through 0.8% alkaline agarose gels in 30 mM NaOH and 2 mM EDTA for 16 h at 25 V. Gels were fixed with 5% cold trichloroacetic acid and then dried onto chromatography paper (Whatman) and autoradiographed with Amerhsam Hyperfilm-MP (GE Healthcare). Gel images were scanned using a Typhoon phosphorimager (GE Healthcare) and were quantified using ImageJ.

### Electrophoretic mobility shift assays

Double-stranded DNA probes for electrophoretic mobility shift assays (EMSA) were generated by PCR using primers (50 bp up 209 and 50 bp down 209) to give 265 bp probes. A full list of primers and plasmids used for DNA templates is given in Supplementary Tables 1 and 2.

A total of 62.5 ng gel-purified PCR product (Nucleospin Gel and PCR clean-up, 740609.250 Macherey Nagel) was 5′-end-labeled in a final volume of 10 μl using 0.6 μl T4 PNK (10 U μl^−1^; New England Biolabs, M0201) with 2 μl [gamma-P32]ATP (3,000 Ci mmol^−1^; SRP-301 Hartmann). After 1 h on ice, 40 μl 10 mM HEPES-KOH pH 7.6, 1 mm EDTA was added, and unincorporated nucleotides were removed by passing through 2× microspin G-50 columns (Cytiva, GE27-5330-02), pre-spun for 1 min at 735*g*, followed by elution for 2 min at 735*g*.

For each EMSA reaction, 12 fmol of DNA probe was used (assuming 80% elution losses per column). Reaction components were added on ice to give a final concentration of 25 mM HEPES-KOH pH 7.6, 10 mM MgOAc, 100 mM NaOAc, 0.02% NP-40, 5 mM DTT, 80 mM KCl, 5% glycerol, 2 μg ml^−1^ poly dI-dC and 5 mM ATP. Lastly, 100 fmol of purified ORC was added as required, and the reactions were incubated for 30 min at 30 °C in a thermomixer at 800 rpm. After 30 min, the samples were placed on ice, and 2 μl loading dye (Purple gel loading dye, no SDS; New England Biolabs, B7025) was added before loading 12 μl onto a 1.5% 15 × 15 cm 100 ml 0.5× TB agarose gel. The gel was run for 1 h at 200 V (4 °C) before fixation for 30 min in 5% TCA and drying on a vacuum gel dryer at 55 °C for 1 h (Hoefer, GD2000). After overnight exposure to a storage phosphor screen (GE Healthcare, BAS-IP MS 2025 E), the signal was detected using a Typhoon FLA7000 phosphoimager.

### Induction of re-replication

Cells were grown overnight in medium lacking methionine and containing 2% raffinose (1× YNB, 1× CSM-Met; Formedium, DCS0111). At a density of about 1 × 10^7^ cells per ml, 50 ng ml^−1^ alpha factor was added to arrest the cell growth for 3 h. Cells were spun down and switched to YPRaff medium with alpha factor for 1 h. Cells were washed twice with YPRaff media (1× with YPRaff and 1× with YPRaff + 50 μg ml^−1^ Pronase E (Sigma-Aldrich, 1074330001). Cells were resuspended in YPRaff medium containing 100 μg ml^−1^ Pronase E with 5 μg ml^−1^ nocodazole for 3 h. Cells were then divided into two and 2% of glucose or galactose was added, respectively, to induce Cdc6∆NT for 3 h in the presence of galactose only. Cells were collected and lysed with lysis buffer (2% Triton, 1% SDS, 100 mM NaCl, 10 mM Tris pH 8.0, 1 mM EDTA and protease inhibitor (AEBSF, Leupeptin, Pepstatin A)). Glass beads and phenol/chloroform were added together and tubes were vortexed for 1 min. The supernatant was treated with RNases at 25 °C for 30 min and then extracted with phenol/chloroform and ethanol-precipitated. The DNA was used in qPCR. A total of 9 μl of qPCR reaction was set up in 384-well plates with 4.5 μl of 2× FastStart Universal SYBR Green Master mix ROX (Roche), ~0.28 μM of primers (set 1: CTL141 + CTL142 for re-replication region; set 2: CTL143 + CTL144 for internal control) for 10 min at 95 °C and then 40 cycles of 10 s at 95 °C and 30 s at 60 °C. Each DNA sample was first evaluated for the linearity of PCR by performing serial dilutions. The fold change of threshold cycle (Ct) was determined using the 2^−^^∆∆Ct^ method^49^, where ∆∆Ct = (Ct_re-replication region_ – Ct_internal control_)_galactose_ – (Ct_re-replication region_ – Ct_internal control_)_glucose_.

### Nucleosome-flanked origins

Nucleosome-flanked origins of replication were prepared with purified yeast histone octamers as previously described^11^ on the DNA substrates of N-ARS1-N or N-90bp-N. Plasmids containing N-ARS1-N and N-90bp-N were synthesized by Eurofins and used as a template for PCR to produce linear DNA substrates using the primers NCP F and NCP R. Origin substrates were amplified using standard PCR protocols and were purified by anion exchange chromatography using a 1 ml RESOURCE Q column (GE Healthcare). Peak fractions were ethanol-precipitated and resuspended in TE buffer.

### Nucleosome assembly

Purified origin DNA substrates were combined with soluble histone octamers to form nucleosomes by salt deposition^11,50^. Nucleosome reconstitution was optimized in small-scale titrations, and the products were checked by 4% native PAGE.

### Negative-stain EM MCM loading assays

MCM loading experiments, negative-stain sample preparation, imaging and analysis were carried out as previously described^11^, with minor modifications (see below).

### Sample preparation

Nucleosome-flanked ARS1/90 bp origin substrates (7.5 nM) were incubated with WT or mutant ORC (20 nM), Cdc6 (20 nM) and MCM–Cdt1 (40 nM) with mixing (1,250 rpm) in EM buffer (25 mM HEPES pH 7.6, 10 mM MgOAc, 100 mM NaOAc, 0.02% NP-40, 80 mM KCl, 1 mM DTT, 5% glycerol) and 2 mM ATP in a final reaction volume of 20 μl. In experiments testing CDK-phosphorylated ORC, ORC was pre-phosphorylated for 10 min before Sic1 was added for 5 min to inhibit further CDK activity. Negative-stain grids were prepared after 2 min (MO detection) and 10 min (DH detection) incubation at 24 °C. In all cases, samples were diluted fivefold before making grids.

### Grid preparation

Samples were applied to glow-discharged 300-mesh copper grids with carbon film (EM Resolutions). A 3 µl aliquot of sample was applied to each grid and incubated for 1 min. Staining was performed with four drops of 40 μl 2% uranyl acetate, and the grids were blotted to remove excess stain.

### Data collection

Micrographs were collected on a Tecnai LaB6 G^2^ Spirit transmission electron microscope (FEI) operating at 120 keV, with a 2K × 2K GATAN Ultrascan 100 camera. Images were recorded at a nominal magnification of 30K (3.45 Å pixel size) and a defocus range of ~0.5–2 μm.

### Image processing

All datasets were processed in Relion (v.3.1)^51^ using a common processing pipeline. The contrast transfer function (CTF) of each micrograph was estimated using Gctf (v.1.06)^52^. Particles were picked with Topaz^53^ using a model that had been pre-trained to pick MCM–Cdt1, ORCs, MOs and MCM-DHs in negative-stain EM data. Particles were initially extracted with a 128-pixel box, rescaled to 64 pixels and subjected to two rounds of reference-free 2D classification. All MCM-containing particles were re-extracted with a 128-pixel box without rescaling. The particles were subjected to a further round of 2D classification. From this classification, particles that contributed to classes containing the MO and DH intermediates were selected and further classified, as required. Finally, particles contributing to classes that contained an MCM (total MCMs, MOs and DHs) were quantified from the appropriate 2D classification. The proportion of MCM-containing particles contributing to each intermediate state was plotted using Prism.

### ReconSil

Datasets for ReconSil images were processed together in Relion (v.3.1)^51^. CTF estimation was performed using Gctf (v.1.06)^52^. Particles were picked with Topaz^53^ using models pre-trained to pick MCM-containing particles (as above), ORCs and nucleosomes. Particle picks were combined, duplicates removed and extracted particles were subjected to multiple rounds of reference-free 2D classification. ReconSil micrographs were generated using the command-line tool ‘relion_particle_reposition’ in Relion (v.3.1) to overlay particles in the raw micrographs with the 2D averages that those particles contributed to. Individual origins were extracted from raw and ReconSil micrographs (320-pixel box size). Representative examples were selected from fully reconstituted origins where confident assignment of co-localization to the same origin could be made (as previously described^11^).

### Cryo-EM of DNA-bound SHs and DHs loaded by ORC phosphorylated on Orc2

Image acquisition, refinement and validation statistics for the SH and DH structures below can be found in Table 1.

### Sample preparation

Samples were prepared as described above for the negative-stain EM experiments with the following modifications:

Mutant ORC (Orc6-4A; 40 nM) was phosphorylated by CDK (10 nM) for 10 min in CDK buffer (25 mM HEPES pH 7.6, 10 mM MgOAc, 100 mM NaOAc, 80 mM KCl, 1 mM DTT, 2 mM ATP), before Sic1 (30 nM) was added to inhibit further CDK activity. Nucleosome-flanked ARS1 origin substrates (15 nM) were incubated with pre-phosphorylated Orc6-4A mix (as above), Cdc6 (40 nM) and MCM–Cdt1 (80 nM), with mixing (1,250 rpm) in cryo-EM buffer (25 mM HEPES pH 7.6, 10 mM MgOAc, 100 mM NaOAc, 80 mM KCl, 1 mM DTT) and 2.2 mM ATP in a final reaction volume of 50 μl. The reaction was incubated at 30 °C for 30 min before being used to prepare cryo-EM grids. Next, 4 μl of sample was applied to fresh graphene-oxide coated 300-mesh UltrAuFoil R1.2/1.3 grids^54^ and incubated for 30 s before vitrification using a Vitrobot Mark IV (Thermo Fisher) cooled to 10 °C with 100% humidity. Grids were blotted for 5 s and plunged into liquid ethane.

### Cryo-EM data collection

Data were collected on a Titan Krios EM equipped with a K2 Summit direct electron detector (Gatan) at the Francis Crick Institute (Structural Biology STP).

### Cryo-EM image processing

#### SH and DH

**Extended Data Fig. 8:**
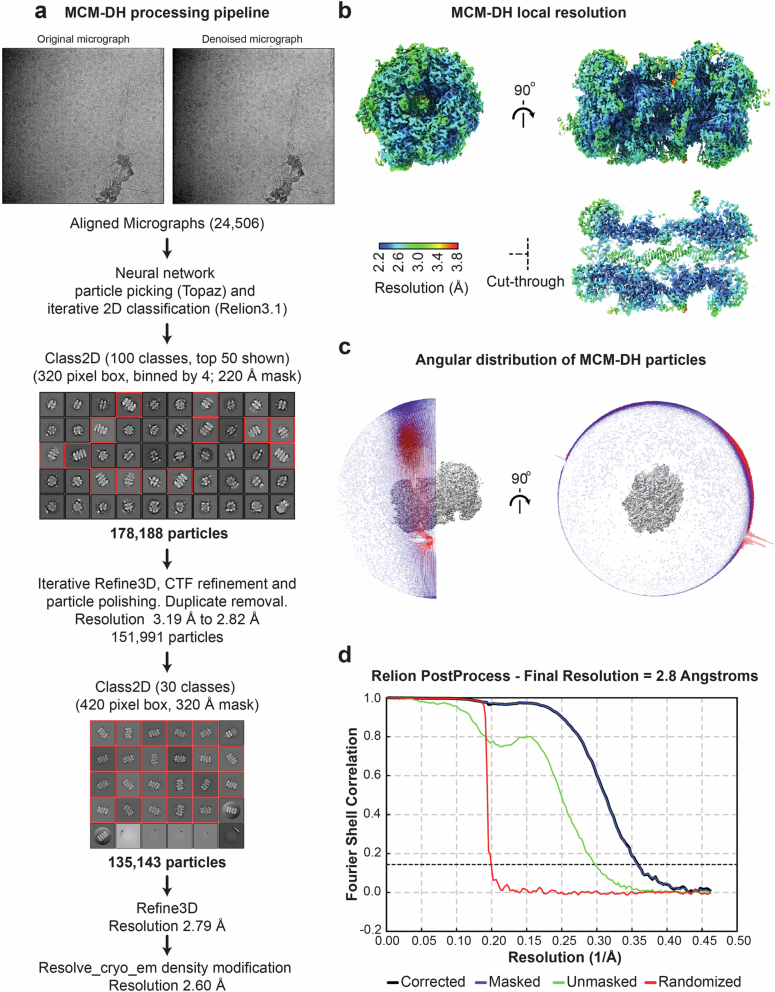
DH cryo-EM data processing. **a**. Cryo-EM data processing pipeline for the DH. **b**. Final DH EM density map following density modification with resolve_cryo_em^56^, coloured according to the local resolution. **c**. Angular distribution plot. **d**. Fourier shell correlation (FSC) plot obtained from Relion PostProcess.

**Extended Data Fig. 9:**
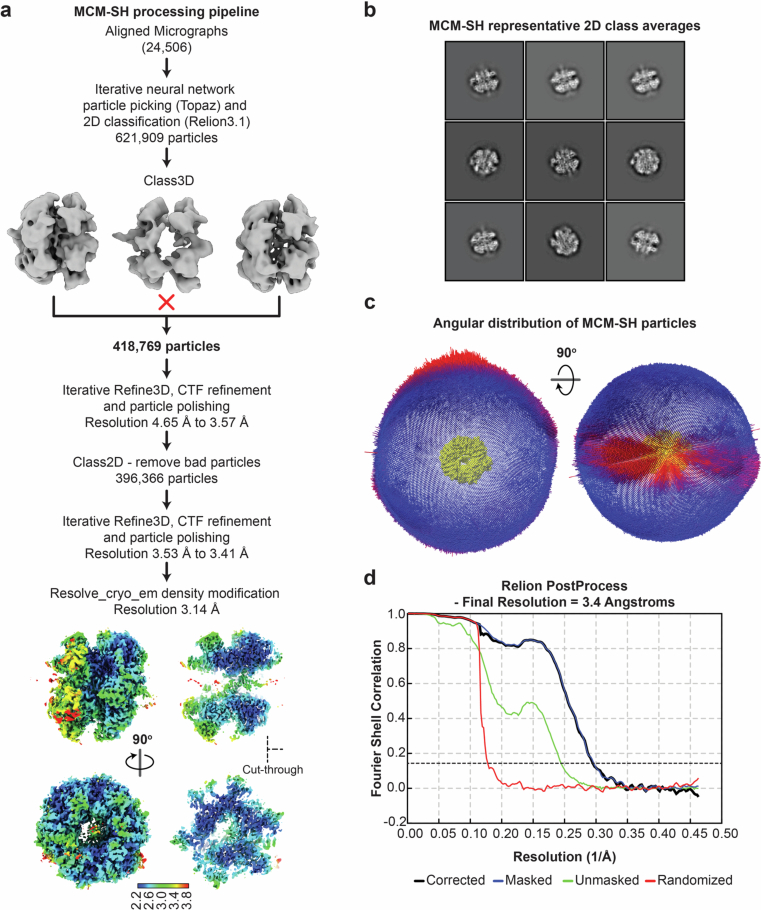
SH data cryo-EM processing. **a**. Cryo-EM data processing pipeline for the SH, including views of the resulting 3D density modified map coloured according to local resolution. **b**. Representative 2D classes for the SH. **c**. Angular distribution plot. **d**. Fourier shell correlation (FSC) plot obtained from Relion PostProcess.

Image processing was performed in RELION (v.3.1)^51^ (Extended Data Figs. 8 & 9). Movie stacks were aligned and motion-corrected using MotionCor2 (ref. ^55^), retaining all frames. The CTF of each micrograph was estimated using Gctf v1.06 (ref. ^52^). Particles were automatically picked using Topaz^53^. First, particles were picked using a general Topaz model (scale factor, 4; model, resnet8_u32; radius, 20). These particles were extracted (320-pixel box, rescaled to 80 pixels) and subjected to multiple rounds of reference-free 2D classification to isolate SH and DH particles for subsequent processing.

High-quality DH particles were subjected to iterative rounds of 3D refinement, particle polishing (including increasing the box size to 420 pixels), CTF refinement and a final round of 2D classification, yielding the final EM map at an average resolution of 2.8 Å, reconstructed from 135,143 MCM-DH particles. The final RELION map was subject to density modification using resolve_cryo_em in Phenix 1.19.2 (ref. ^56^) to generate a 2.6 Å map used for model building (Extended Data Fig. 5).

SH particles obtained from 2D classification were used for Topaz training (scale factor, 4; cnn_model, resnet8; radius, 3). SH particles were picked using the new Topaz model (selection threshold, −2), extracted (280-pixel box, rescaled to 70 pixels) and subjected to two rounds of reference-free 2D classification. SH particles were re-extracted (280-pixel box, unbinned) and classified into three classes using a 30 Å filtered reference from an ab initio reconstruction in CryoSPARC^57^. High-quality SH particles were subjected to iterative rounds of particle polishing (including increasing the box size to 420 pixels), CTF refinement, masked 3D refinement and a final round of 2D classification (without alignment), yielding the final EM map at an average resolution of 3.4 Å, reconstructed from 396,366 SH particles. The final RELION map was subject to density modification using resolve_cryo_em in Phenix 1.19.2 (ref. ^56^) to generate a 3.1 Å map used for model building (Extended Data Fig. 5).

### Molecular modeling

#### DH

Molecular modeling of the DH complex was performed using a density-modified map generated using resolve_cryo_em. A published DH model (PDB 7P30)^58^ was used as an initial model and rigid-body docked into the new DH EM map. The resulting model was subject to manual modification in Coot^59^ and real-space refinement in Phenix 1.19.2 (ref. ^60^). All figures were generated using UCSF ChimeraX^61^.

#### SH

Molecular modeling of the SH complex was performed using a density-modified map generated using resolve_cryo_em. An initial model of a loaded SH was extracted from the higher-resolution DH model (this study) and rigid-body docked into the SH EM map. The resulting model was subject to iterative rounds of manual modification in Coot^59^ and real-space refinement in Phenix 1.19.2 (ref. ^60^). All figures were generated using UCSF ChimeraX^61^.

### Orc2 IDR AlphaFold2 modeling

The MO interaction interface, consisting of *Saccharomyces* *cerevisiae* Orc2 (P32833), Orc3 (p54790), Orc5 (P50874, amino acids 319–479), Orc6 (P38826), Mcm2 (P29469, amino acids 178–457), Mcm5 (P29496 amino acids 1–336) and Mcm6 (P53091, amino acids 88–463), was modeled using AlphaFold2 (2.3.1)^14^ implemented in AlphaPulldown (0.30.0)^62^. The five resulting models positioned the Orc2 IDR amino acids 190–231 in the same location, wrapping around the Orc6 TFIIB-B domain. Orc3 (amino acids 270–419) in the top-ranked model was aligned with the corresponding sequence in the previously determined MO structure (PDB 6RQC)^11^ using the matchmaker tool in ChimeraX (v.1.6)^60^. The Orc2 IDR (amino acids 190–231) was fit as a rigid body into the previously unassigned density in the MO map using the ‘Fit in Map’ tool in ChimeraX. The entire MO model, with the Orc2 IDR, was subjected to a single round of real-space refinement in Phenix (v.1.21.2)^60^. Figure 1c and Extended Data Fig. 1 (AlphaFold2 figure) display EMready^45^ density-modified EM maps obtained from the multibody refinement of the MO intermediate. Similar AlphaFold2 predictions of the ORC subunits alone, or the ORC subunits with the complete N-terminal domains of Mcm2–7, yielded almost identical predictions for the interaction of the Orc2 IDR with Orc6.

### Cryo-EM of MO formation assays on 90 bp synthetic origins

#### Sample preparation

Samples were prepared as described above for the cryo-EM experiments with the following modifications:

Nucleosome-flanked 90 bp origin substrates (15 nM) were incubated with WT ORC or Orc2ΔN (40 nM), Cdc6 (40 nM) and MCM–Cdt1 (80 nM), with mixing (1,250 rpm) in cryo-EM buffer (25 mM HEPES pH 7.6, 10 mM MgOAc, 100 mM NaOAc, 80 mM KCl, 1 mM DTT) and 2.2 mM ATP in a final reaction volume of 20 μl. The reaction was incubated at 24 °C for 2 min before cryo-EM grid preparation. Then, 4 μl of sample was applied to fresh graphene-oxide-coated 300-mesh UltrAuFoil R1.2/1.3 grids^54^ and incubated for 30 s before vitrification using a Vitrobot Mark IV (Thermo Fisher) cooled to 10 °C with 100% humidity. Grids were blotted for 5 s and plunged into liquid ethane.

### Cryo-EM data collection

Data were collected at the Francis Crick Institute (Structural Biology STP) on a 300 kV Titan Krios electron microscope equipped with a Falcon4i direct electron detector (Thermo Fisher Scientific). Movies were recorded at a magnification of ×130,000 (pixel size, 1.08 Å) with an electron exposure (e^−^ Å^−2^) of 32.5 and a defocus range of 1–5 μm. In total, 24,315 movies were collected for the Orc2ΔN dataset and 21,370 for the WT ORC dataset.

### Cryo-EM image processing

Movie stacks were aligned and motion-corrected using MotionCor2 (ref. ^55^), retaining all frames. All downstream processing was performed in CryoSPARC (v.4.4.0). The CTF of each micrograph was estimated using Patch CTF estimation. Particles were automatically picked using the Blob Picker using the default parameters (minimum/maximum particle diameters, 150 Å/250 Å; minimum separation distance, 0.7). Micrographs with a poor CTF fit, no graphene oxide (empty holes) or ice contamination were removed, yielding final micrograph stacks of 18,414 micrographs (Orc2ΔN) and 18,156 (WT ORC). Particles were initially extracted (560-pixel box, rescaled to 140 pixels) and subjected to multiple rounds of reference-free 2D classification to isolate MCM-containing particles. A class depicting the MCM single hexamer was selected and used for subsequent Template Picking (particle diameter, 200 Å), and extracted particles were subjected to reference-free 2D classification, as above. MCM-containing particle stacks from the Blob and Template pickers were merged and duplicate particles removed before final 2D classifications with unbinned particles (560-pixel box).

High-quality DH particles were additionally subjected to Ab Initio 3D reconstruction and non-uniform 3D refinement^63^, yielding EM maps at average resolutions of 2.96 Å (WT ORC) and 3.08 Å (Orc2ΔN), reconstructed from 149,543 and 137,314 MCM-DH particles, respectively.

### Simulating MCM-DH formation efficiency from two diffusing MCM-SHs

We model the separation-dependent MCM-DH formation efficiency using stochastic simulations of one-dimensional diffusion of MCM-SHs sliding along the DNA. MCM-DH formation efficiency is computed from the probability of collision of two diffusing MCM-SHs given by the intersection of their diffusion trajectories. The simulation consists of four key steps: MCM-SH loading, MCM-SH diffusion, MCM-SH dissociation and MCM-DH formation.

#### MCM-SH loading

The simulation takes place on a DNA substrate consisting of two origin sequences in a head-to-head orientation, with the 5′ ends of the ORC binding site pointed towards each other. The separation between the two origins is defined as the distance between their 5′ ends of the ORC binding sites, as shown in Extended Data Fig. 7b. ORC binds to an origin sequence with an overhang of 6 bp ahead of the ORC binding site^15,64^ (Extended Data Fig. 7c). This ORC complex can then load an MCM-SH ahead of itself, which occupies 35 bp on the DNA substrate^65^. This implies that the loading of one MCM-SH complex onto the DNA occupies at least 41 bp (6 bp ORC overhang + 35 bp MCM-SH) past the origin on the DNA substrate. The simulation is initialized with both ORC sites loaded with MCM-SHs with N-termini pointing towards each other.

#### MCM-SH diffusion

Given that MCM-SH sliding is independent of ATP hydrolysis, we assume that its diffusion occurs at thermal equilibrium and is therefore not driven by the input of external energy. In such conditions, the displacement of MCM-SH per unit time follows a Gaussian distribution given by:

1 $$P\left(x,\Delta t\right)=\frac{1}{\sqrt{4\pi D\Delta t}}\,{e}^{-\frac{{(x-{x}_{0})}^{2}}{4D\Delta t}}$$

where $x$ is the position of the MCM-SH, $D$ is the diffusion coefficient, $\Delta t$ is the duration of the time step and ${x}_{0}$ is the initial position. After initialization of the simulation, the displacement of MCM-SH in the following time step is determined by sampling this probability distribution using the value of MCM-SH diffusion constants measured from single-molecule fluorescence tracking experiments^31,32^.

2 $${x}_{n+1}={x}_{n}+P(x,\Delta t)$$

3 $${x}_{n+1}={x}_{n}+\sqrt{2D\Delta t}\times{\rm{randn}}(\,)$$

where ‘randn()’ is the MATLAB function to generate normally distributed random numbers. The time step of the simulation is chosen such that the mean squared displacement (MSD) of MCM-SH for the given value of diffusion constant is less than 1 bp of the DNA.

4 $${\rm{MSD}}=\, < {\left(x\left(\Delta t\right)-{x}_{0}\right)}^{2} > \,=2D\Delta t$$

Thus, the time step of the simulation was chosen to be 10 μs.

#### MCM-SH dissociation from the DNA

Once loaded onto the DNA substrate, MCM-SHs dissociate from the DNA with an experimentally measured half-life of 3 min (Fig. 5b). We implement this phenomenon in our simulations by assigning a dissociation probability to each loaded MCM-SH onto the DNA. The dissociation probability is computed using the exponential distribution that satisfies the experimentally measured half-life:

5 $${\tau }_{{\rm{MCM}}\,{\rm {SH}}}=\,-\frac{{\tau }_{{\rm{MCM}}\,{\rm {SH}}}^{1/2}}{{\log }_{{\rm{e}}}\left(0.5\right)}$$

6 $${P}_{\rm{dissociation}}=\frac{1}{{\tau }_{\rm{MCM}\,\rm{SH}}}\,{{\rm{e}}}^{-\frac{t}{{\tau }_{\rm{MCM}\,\rm{SH}}}}$$

where ${\tau }_{\rm{MCM}\,\rm{SH}}^{1/2}$ is the experimentally measured MCM-SH half-life and $t$ is the total time spent by MCM-SH on the DNA since its loading. At every time step, a random number, *R*, is sampled from a uniform distribution using the MATLAB function rand(). If *R* is smaller than ${P}_{\rm{dissociation}}$, the corresponding MCM-SH is said to be dissociated from the DNA, and the simulation is stopped as MCM-DH formation is no longer possible.

#### MCM-DH formation

To successfully form an MCM-DH, the two loaded MCM-SHs must collide with each other while diffusing along the DNA. We define a collision event as the first instance when the distance between the N-termini of both MCM-SHs is ≤0 bp during the simulation. The time duration from the start of the simulation to the time step corresponding to the collision event is referred to as the collision time, which also corresponds to the first-passage time of the collision process. The probability of MCM-DH formation is thus the probability of collision of two MCM-SHs loaded onto the DNA, which depends on the separation between the ORC binding sites, the availability of the binding site on the DNA for the second MCM-SH, the diffusion constants of the MCM-SHs, the half-life of MCM-SH on the DNA and the total time for the simulation.

#### Simulation results

The diffusion coefficient of MCM onto the DNA has been observed to be 800 ± 200 bp^2^ s^−1^ from single-molecule fluorescence tracking experiments^32^. The half-life of MCM-SH on the DNA is measured to be 3 min (Fig. 5b). Using these values as our simulation parameters, we quantify the dynamics of MCM-DH formation as a function of the separation between the ORC loading sites as follows. For each case of separation, we first quantify the number of simulated trajectories that contain a collision event between two MCM-SHs and measure the time between the start of the simulation to the collision event, hereafter referred to as collision time (${t}_{c}$) Extended Data Fig. 7b. The probability distribution of collision times, $P({t}_{c})$, is generated from all the iterations (Extended Data Fig. 7c). The cumulative sum of this distribution, given by $C\left(t\right)=\mathop{\sum }\nolimits_{{t}_{c}=0}^{t}P({t}_{c})$, refers to the probability of collisions occurring before time $t$, which is, by definition, the probability of MCM-DH formation within time $t$ (Extended Data Fig. 7d). Computing $C(t)$ for various separations between ORC binding sites at a fixed time (20 min) determined by the incubation time in bulk experiments (Fig. 5d) allows us to compare the relative MCM-DH formation efficiencies on the corresponding DNA substrates (Extended Data Fig. 7e).

We observe that the relative MCM-DH formation efficiency has a maximum at separation of 82 bp between the ORC binding sites, which corresponds to the case when MCM-SHs are loaded closest to each other onto the DNA (ORC overhang + MCM-SH + MCM-SH + ORC overhang = 6 + 35 + 35 + 6 = 82 bp). At separations greater than 82 bp, the MCM-DH formation efficiency decreases with increasing separation (and shows a good agreement with exponential trend) as both MCM-SHs loaded onto the DNA must diffuse along the DNA and collide with each other before either or both of them dissociate from the DNA.

### Reporting summary

Further information on research design is available in the Nature Portfolio Reporting Summary linked to this article.

## Online content

Any methods, additional references, Nature Portfolio reporting summaries, source data, extended data, supplementary information, acknowledgements, peer review information; details of author contributions and competing interests; and statements of data and code availability are available at 10.1038/s41594-025-01591-9.

## Supplementary information

Supplementary Information Supplementary Tables 1–3, sequences of plasmids.

Reporting Summary

Supplementary Video 1 Two MCM single hexamers diffusing via rotation along B-form DNA. By using the first steric clash between hexamers as the end point of rotational diffusion, an inter-ring register is obtained which appears close to the register observed in the MCM double hexamer.

Supplementary Code 1 MCM diffusion simulations.

## Source data

Source Data Fig. 1 Fig. 1d: unprocessed gel.

Source Data Fig. 1 Fig. 1e and f: statistical source data; Fig. 1g: negative-stain EM.

Source Data Fig. 2 Fig. 2b,f,g: statistical source data.

Source Data Fig. 2 Fig. 2d: Unprocessed gel.

Source Data Fig. 3 Fig. 3b,d,e: statistical source data.

Source Data Fig. 3 Fig. 3a,c: unprocessed gel; Fig. 3b(ii), Fig. 3f(i) and Fig. 3g: negative-stain EM.

Source Data Fig. 5 Fig. 5a,b,e,f,g and ED_Fig. 6a: statistical source data.

Source Data Extended Data Fig. 2 ED_Fig. 2e: statistical source data.

Source Data Extended Data Fig. 2 ED_Fig. 2a,d: unprocessed gel.

Source Data Extended Data Fig. 6 ED_Fig. 6d,e: statistical source data.

Source Data Extended Data Fig. 6 ED_Fig. 6c: unprocessed western blot.

## Data Availability

The data supporting this study are available within the paper, its Supplementary Information and Source Data files. Atomic model coordinates and cryo-EM maps have been deposited in the PDB and Electron Microscopy Data Bank (EMDB), under accession codes PDB 8RIF and EMD-19186 (DH), PDB 8RIG and EMD-19187 (SH) and PDB 9I3I (MO with Orc2 IDR). Access to unprocessed electron microscopy datasets can be provided by the corresponding author upon reasonable request. Source data are provided with this paper.

## References

[1] Lin, Y. C. & Prasanth, S. G. Replication initiation: implications in genome integrity. *DNA Repair (Amst.)* **103**, 103131 (2021). 33992866 10.1016/j.dnarep.2021.103131 PMC8296962

[2] Hu, Y. & Stillman, B. Origins of DNA replication in eukaryotes. *Mol. Cell* **83**, 352–372 (2023). 36640769 10.1016/j.molcel.2022.12.024 PMC9898300

[3] Costa, A. & Diffley, J. F. X. The initiation of eukaryotic DNA replication. *Annu. Rev. Biochem.* **91**, 107–131 (2022). 35320688 10.1146/annurev-biochem-072321-110228

[4] Palzkill, T. G. & Newlon, C. S. A yeast replication origin consists of multiple copies of a small conserved sequence. *Cell* **53**, 441–450 (1988). 3284655 10.1016/0092-8674(88)90164-x

[5] Coster, G. & Diffley, J. F. X. Bidirectional eukaryotic DNA replication is established by quasi-symmetrical helicase loading. *Science* **357**, 314–318 (2017). 28729513 10.1126/science.aan0063 PMC5608077

[6] Bell, S. P. & Stillman, B. ATP-dependent recognition of eukaryotic origins of DNA replication by a multiprotein complex. *Nature* **357**, 128–134 (1992). 1579162 10.1038/357128a0

[7] Rao, H. & Stillman, B. The origin recognition complex interacts with a bipartite DNA binding site within yeast replicators. *Proc. Natl Acad. Sci. USA* **92**, 2224–2228 (1995). 7892251 10.1073/pnas.92.6.2224 PMC42456

[8] Rowley, A., Cocker, J. H., Harwood, J. & Diffley, J. F. X. Initiation complex assembly at budding yeast replication origins begins with the recognition of a bipartite sequence by limiting amounts of the initiator, ORC. *EMBO J.* **14**, 2631–2641 (1995). 7781615 10.1002/j.1460-2075.1995.tb07261.x PMC398377

[9] Ticau, S. et al. Mechanism and timing of Mcm2–7 ring closure during DNA replication origin licensing. *Nat. Struct. Mol. Biol.* **24**, 309–315 (2017). 28191892 10.1038/nsmb.3375 PMC5336523

[10] Ticau, S., Friedman, L. J., Ivica, N. A., Gelles, J. & Bell, S. P. Single-molecule studies of origin licensing reveal mechanisms ensuring bidirectional helicase loading. *Cell* **161**, 513–525 (2015). 25892223 10.1016/j.cell.2015.03.012 PMC4445235

[11] Miller, T. C. R., Locke, J., Greiwe, J. F., Diffley, J. F. X. & Costa, A. Mechanism of head-to-head MCM double-hexamer formation revealed by cryo-EM. *Nature* **575**, 704–710 (2019). 31748745 10.1038/s41586-019-1768-0 PMC6887548

[12] Gupta, S., Friedman, L. J., Gelles, J. & Bell, S. P. A helicase-tethered ORC flip enables bidirectional helicase loading. *eLife*10.7554/eLife.74282 (2021). 10.7554/eLife.74282 PMC8828053 34882090

[13] Amasino, A. L., Gupta, S., Friedman, L. J., Gelles, J. & Bell, S. P. Regulation of replication origin licensing by ORC phosphorylation reveals a two-step mechanism for Mcm2–7 ring closing. *Proc. Natl Acad. Sci. USA* **120**, e2221484120 (2023). 37428921 10.1073/pnas.2221484120 PMC10629557

[14] Jumper, J. et al. Highly accurate protein structure prediction with AlphaFold. *Nature* **596**, 583–589 (2021). 34265844 10.1038/s41586-021-03819-2 PMC8371605

[15] Li, N. et al. Structure of the origin recognition complex bound to DNA replication origin. *Nature* **559**, 217–222 (2018). 29973722 10.1038/s41586-018-0293-x

[16] Evans, R. et al. Protein complex prediction with AlphaFold-Multimer. Preprint at 10.1101/2021.10.04.463034 (2022).

[17] Perkins, G., Drury, L. S. & Diffley, J. F. X. Separate SCF^CDC4^ recognition elements target Cdc6 for proteolysis in S phase and mitosis. *EMBO J.* **20**, 4836–4845 (2001). 11532947 10.1093/emboj/20.17.4836 PMC125267

[18] Drury, L. S., Perkins, G. & Diffley, J. F. X. The cyclin-dependent kinase Cdc28p regulates distinct modes of Cdc6p proteolysis during the budding yeast cell cycle. *Curr. Biol.* **10**, 231–240 (2000). 10712901 10.1016/s0960-9822(00)00355-9

[19] Drury, L. S., Perkins, G. & Diffley, J. F. X. The Cdc4/34/53 pathway targets Cdc6p for proteolysis in budding yeast. *EMBO J.* **16**, 5966–5976 (1997). 9312054 10.1093/emboj/16.19.5966 PMC1170227

[20] Elsasser, S., Chi, Y., Yang, P. & Campbell, J. L. Phosphorylation controls timing of Cdc6p destruction: a biochemical analysis. *Mol. Biol. Cell* **10**, 3263–3277 (1999). 10512865 10.1091/mbc.10.10.3263 PMC25589

[21] Moses, A. M., Liku, M. E., Li, J. J. & Durbin, R. Regulatory evolution in proteins by turnover and lineage-specific changes of cyclin-dependent kinase consensus sites. *Proc. Natl Acad. Sci. USA* **104**, 17713–17718 (2007). 17978194 10.1073/pnas.0700997104 PMC2077061

[22] Liku, M. E., Nguyen, V. Q., Rosales, A. W., Irie, K. & Li, J. J. CDK phosphorylation of a novel NLS–NES module distributed between two subunits of the Mcm2–7 complex prevents chromosomal rereplication. *Mol. Biol. Cell* **16**, 5026–5039 (2005). 16093348 10.1091/mbc.E05-05-0412 PMC1237101

[23] Nguyen, V. Q., Co, C., Irie, K. & Li, J. J. Clb/Cdc28 kinases promote nuclear export of the replication initiator proteins Mcm2–7. *Curr. Biol.* **10**, 195–205 (2000). 10704410 10.1016/s0960-9822(00)00337-7

[24] Labib, K., Diffley, J. F. X. & Kearsey, S. E. G1-phase and B-type cyclins exclude the DNA-replication factor Mcm4 from the nucleus. *Nat. Cell Biol.* **1**, 415–422 (1999). 10559985 10.1038/15649

[25] Tanaka, S. & Diffley, J. F. X. Interdependent nuclear accumulation of budding yeast Cdt1 and Mcm2–7 during G1 phase. *Nat. Cell Biol.* **4**, 198–207 (2002). 11836525 10.1038/ncb757

[26] Yeeles, J. T., Deegan, T. D., Janska, A., Early, A. & Diffley, J. F. X. Regulated eukaryotic DNA replication origin firing with purified proteins. *Nature* **519**, 431–435 (2015). 25739503 10.1038/nature14285 PMC4874468

[27] Frigola, J., Remus, D., Mehanna, A. & Diffley, J. F. X. ATPase-dependent quality control of DNA replication origin licensing. *Nature* **495**, 339–343 (2013). 23474987 10.1038/nature11920 PMC4825857

[28] Chen, S. & Bell, S. P. CDK prevents Mcm2–7 helicase loading by inhibiting Cdt1 interaction with Orc6. *Genes Dev.* **25**, 363–372 (2011). 21289063 10.1101/gad.2011511 PMC3042159

[29] Nguyen, V. Q., Co, C. & Li, J. J. Cyclin-dependent kinases prevent DNA re-replication through multiple mechanisms. *Nature* **411**, 1068–1073 (2001). 11429609 10.1038/35082600

[30] Richardson, C. D. & Li, J. J. Regulatory mechanisms that prevent re-initiation of DNA replication can be locally modulated at origins by nearby sequence elements. *PLoS Genet.* **10**, e1004358 (2014). 24945837 10.1371/journal.pgen.1004358 PMC4063666

[31] Sanchez, H. et al. DNA replication origins retain mobile licensing proteins. *Nat. Commun.* **12**, 1908 (2021). 33772005 10.1038/s41467-021-22216-x PMC7998030

[32] Zhang, A., Friedman, L. J., Gelles, J. & Bell, S. P. Changing protein-DNA interactions promote ORC binding-site exchange during replication origin licensing. *Proc. Natl Acad. Sci. USA* **120**, e2305556120 (2023). 37463200 10.1073/pnas.2305556120 PMC10372627

[33] Frigola, J. et al. Cdt1 stabilizes an open MCM ring for helicase loading. *Nat. Commun.* **8**, 15720 (2017). 28643783 10.1038/ncomms15720 PMC5490006

[34] Sanchez, H. et al. A chromatinized origin reduces the mobility of ORC and MCM through interactions and spatial constraint. *Nat. Commun.* **14**, 6735 (2023). 37872142 10.1038/s41467-023-42524-8 PMC10593741

[35] Hizume, K., Kominami, H., Kobayashi, K., Yamada, H. & Araki, H. Flexible DNA path in the MCM double hexamer loaded on DNA. *Biochemistry* **56**, 2435–2445 (2017). 28459551 10.1021/acs.biochem.6b00922

[36] Donovan, S., Harwood, J., Drury, L. S. & Diffley, J. F. X. Cdc6p-dependent loading of Mcm proteins onto pre-replicative chromatin in budding yeast. *Proc. Natl Acad. Sci. USA* **94**, 5611–5616 (1997). 9159120 10.1073/pnas.94.11.5611 PMC20826

[37] Santocanale, C., Sharma, K. & Diffley, J. F. X. Activation of dormant origins of DNA replication in budding yeast. *Genes Dev.* **13**, 2360–2364 (1999). 10500092 10.1101/gad.13.18.2360 PMC317032

[38] Vujcic, M., Miller, C. A. & Kowalski, D. Activation of silent replication origins at autonomously replicating sequence elements near the HML locus in budding yeast. *Mol. Cell. Biol.* **19**, 6098–6109 (1999). 10454557 10.1128/mcb.19.9.6098 PMC84529

[39] Ibarra, A., Schwob, E. & Mendez, J. Excess MCM proteins protect human cells from replicative stress by licensing backup origins of replication. *Proc. Natl Acad. Sci. USA* **105**, 8956–8961 (2008). 18579778 10.1073/pnas.0803978105 PMC2449346

[40] Woodward, A. M. et al. Excess Mcm2–7 license dormant origins of replication that can be used under conditions of replicative stress. *J. Cell Biol.* **173**, 673–683 (2006). 16754955 10.1083/jcb.200602108 PMC2063885

[41] Hu, Y. et al. Evolution of DNA replication origin specification and gene silencing mechanisms. *Nat. Commun.* **11**, 5175 (2020). 33056978 10.1038/s41467-020-18964-x PMC7560902

[42] Lee, C. S. K. et al. Humanizing the yeast origin recognition complex. *Nat. Commun.* **12**, 33 (2021). 33397927 10.1038/s41467-020-20277-y PMC7782691

[43] Parker, M. W. et al. A new class of disordered elements controls DNA replication through initiator self-assembly. *eLife*10.7554/eLife.48562 (2019). 10.7554/eLife.48562 PMC6764820 31560342

[44] Salas-Leiva, D. E. et al. Genomic analysis finds no evidence of canonical eukaryotic DNA processing complexes in a free-living protist. *Nat. Commun.* **12**, 6003 (2021). 34650064 10.1038/s41467-021-26077-2 PMC8516963

[45] He, J., Li, T. & Huang, S. Y. Improvement of cryo-EM maps by simultaneous local and non-local deep learning. *Nat. Commun.* **14**, 3217 (2023). 37270635 10.1038/s41467-023-39031-1 PMC10239474

[46] Yuan, Z. et al. Structural basis of Mcm2–7 replicative helicase loading by ORC-Cdc6 and Cdt1. *Nat. Struct. Mol. Biol.* **24**, 316–324 (2017). 28191893 10.1038/nsmb.3372 PMC5503505

[47] Cameron, G. et al. Sister chromatid cohesion establishment during DNA replication termination. *Science* **384**, 119–124 (2024). 38484038 10.1126/science.adf0224 PMC7615807

[48] Drury, L. S. & Diffley, J. F. X. Factors affecting the diversity of DNA replication licensing control in eukaryotes. *Curr. Biol.* **19**, 530–535 (2009). 19285403 10.1016/j.cub.2009.02.034

[49] Schmittgen, T. D. et al. Quantitative reverse transcription-polymerase chain reaction to study mRNA decay: comparison of endpoint and real-time methods. *Anal. Biochem.* **285**, 194–204 (2000). 11017702 10.1006/abio.2000.4753

[50] Luger, K., Rechsteiner, T. J. & Richmond, T. J. Preparation of nucleosome core particle from recombinant histones. *Methods Enzymol.* **304**, 3–19 (1999). 10372352 10.1016/s0076-6879(99)04003-3

[51] Zivanov, J., Nakane, T. & Scheres, S. H. W. Estimation of high-order aberrations and anisotropic magnification from cryo-EM data sets in RELION-3.1. *IUCrJ* **7**, 253–267 (2020). 32148853 10.1107/S2052252520000081 PMC7055373

[52] Zhang, K. Gctf: real-time CTF determination and correction. *J. Struct. Biol.* **193**, 1–12 (2016). 26592709 10.1016/j.jsb.2015.11.003 PMC4711343

[53] Bepler, T. et al. Positive-unlabeled convolutional neural networks for particle picking in cryo-electron micrographs. *Nat. Methods* **16**, 1153–1160 (2019). 31591578 10.1038/s41592-019-0575-8 PMC6858545

[54] Martin, T. G., Boland, A., Fitzpatrick, A. W. P. & Scheres, S. H. W. Graphene oxide grid preparation. *Figshare*10.6084/m9.figshare.3178669.v1 (2016).

[55] Zheng, S. Q. et al. MotionCor2: anisotropic correction of beam-induced motion for improved cryo-electron microscopy. *Nat. Methods* **14**, 331–332 (2017). 28250466 10.1038/nmeth.4193 PMC5494038

[56] Terwilliger, T. C., Ludtke, S. J., Read, R. J., Adams, P. D. & Afonine, P. V. Improvement of cryo-EM maps by density modification. *Nat. Methods* **17**, 923–927 (2020). 32807957 10.1038/s41592-020-0914-9 PMC7484085

[57] Punjani, A., Rubinstein, J. L., Fleet, D. J. & Brubaker, M. A. cryoSPARC: algorithms for rapid unsupervised cryo-EM structure determination. *Nat. Methods* **14**, 290–296 (2017). 28165473 10.1038/nmeth.4169

[58] Greiwe, J. F. et al. Structural mechanism for the selective phosphorylation of DNA-loaded MCM double hexamers by the Dbf4-dependent kinase. *Nat. Struct. Mol. Biol.* **29**, 10–20 (2022). 34963704 10.1038/s41594-021-00698-z PMC8770131

[59] Casanal, A., Lohkamp, B. & Emsley, P. Current developments in Coot for macromolecular model building of electron cryo-microscopy and crystallographic data. *Protein Sci.* **29**, 1069–1078 (2020). 31730249 10.1002/pro.3791 PMC7096722

[60] Afonine, P. V. et al. Real-space refinement in PHENIX for cryo-EM and crystallography. *Acta Crystallogr. D Struct. Biol.* **74**, 531–544 (2018). 29872004 10.1107/S2059798318006551 PMC6096492

[61] Pettersen, E. F. et al. UCSF ChimeraX: structure visualization for researchers, educators, and developers. *Protein Sci.* **30**, 70–82 (2021). 32881101 10.1002/pro.3943 PMC7737788

[62] Yu, D., Chojnowski, G., Rosenthal, M. & Kosinski, J. AlphaPulldown—a Python package for protein–protein interaction screens using AlphaFold-Multimer. *Bioinformatics*10.1093/bioinformatics/btac749 (2023). 10.1093/bioinformatics/btac749 PMC9805587 36413069

[63] Punjani, A., Zhang, H. & Fleet, D. J. Non-uniform refinement: adaptive regularization improves single-particle cryo-EM reconstruction. *Nat. Methods* **17**, 1214–1221 (2020). 33257830 10.1038/s41592-020-00990-8

[64] Feng, X. et al. The structure of ORC–Cdc6 on an origin DNA reveals the mechanism of ORC activation by the replication initiator Cdc6. *Nat. Commun.* **12**, 3883 (2021). 34162887 10.1038/s41467-021-24199-1 PMC8222357

[65] Noguchi, Y. et al. Cryo-EM structure of Mcm2–7 double hexamer on DNA suggests a lagging-strand DNA extrusion model. *Proc. Natl Acad. Sci. USA*10.1073/pnas.1712537114 (2017). 10.1073/pnas.1712537114 PMC5692578 29078375

[66] Baretic, D. et al. Cryo-EM structure of the fork protection complex bound to CMG at a replication fork. *Mol. Cell* **78**, 926–940.e13 (2020). 32369734 10.1016/j.molcel.2020.04.012 PMC7276988

