# Supplementary material for: Cell cycle regulation has shaped replication origins in budding yeast
Source: Nat Struct Mol Biol. 2025 Jun 30;32(9):1697–707. doi: 10.1038/s41594-025-01591-9 (PMC12440816; doi:10.1038/s41594-025-01591-9)
Supplement: Supplementary file 1 — Supplementary Tables 1–3, sequences of plasmids. [file 41594_2025_1591_MOESM1_ESM.pdf]

nature structural & molecular biology

Supplementary information https://doi.org/10.1038/s41594-025-01591-9

Cell cycle regulation has shaped replication origins in budding yeast

In the format provided by the authors and unedited

Supplementary Information

EcoRI site
XmaI site
Matches to the ORC consensus sequence on the top or bottom strand
LacO
Nucleosome binding site
Abf1
These sequences were cloned in pUC19 at EcoRI and XmaI sites

pCTL40 10bp
gaattcCTCGATTTTTTTATGTTTAGTTTCGCGGACGACGGTTTCGAGGTGGCGAA
AAGCGTCGAAGCGGAACTAAACATAAAAAtAGCGGTGTTCGCCGAGATCGGCTC
GCGCATGGCCGAGTTGAGCGGTTCCAGGCTGGCCACGCAGCATCAGATGGTA
GGCCTCCTGGCGCCGCACCGGCCTCAGCATCCGGTACCTCAGCTGGCCACCG
TCGGCGTCTCGCACGACCACCAGTGCAAGGGTCTGAGCAGCGCCGTCGTGCT
CCTCGGAGTGGAGGCAGCCGAGCGCGACGGTGTGCCCGCCTTCCTGGAGACC
TCCGCGCTCCGCAACCTCCACTTCTACGAGCGGCTCGGCTTCACCGTCACCGC
CGACGTCGAGGTGCCCGAAGGACCGCGCACCTGGTGCATGACCCGCAAGCCC
GGTGCCTGACGCTCGCCACACGACCCGCAGCGCCCGACCGAAAGGAGCGCAC
GAcccggg

pCTL9 12bp
gaattcCTCGATTTTTTTATGTTTAGTTTCGCGGACGACGGTTTCGAGGTGGCGGT
AAAAGCGTCGAAGCGGAACTAAACATAAAAAtAGGTGTTCGCCGAGATCGGCTC
GCGCATGGCCGAGTTGAGCGGTTCCAGGCTGGCCACGCAGCATCAGATGGTA
GGCCTCCTGGCGCCGCACCGGCCTCAGCATCCGGTACCTCAGCTGGCCACCG
TCGGCGTCTCGCACGACCACCAGTGCAAGGGTCTGAGCAGCGCCGTCGTGCT
CCTCGGAGTGGAGGCAGCCGAGCGCGACGGTGTGCCCGCCTTCCTGGAGACC
TCCGCGCTCCGCAACCTCCACTTCTACGAGCGGCTCGGCTTCACCGTCACCGC
CGACGTCGAGGTGCCCGAAGGACCGCGCACCTGGTGCATGACCCGCAAGCCC
GGTGCCTGACGCTCGCCACACGACCCGCAGCGCCCGACCGAAAGGAGCGCAC
GAcccggg

pCTL86 ARS317
gaattcACTAGTACTTAAAAAAACTGTAGTTTCAGTGCAAAAAAGTTTTAACATTAC
GTATCTTGTACCCTTTTTATTGCATATAGAAAGGTCAAATAATCCTTCACATCATG
AAATATAAGCTAAATCGCATTTCTTTTCGTCCACATTTGCAAACAAAACTTTTCAA
TAATAATTTTATAAATAGTATCAATATATATATATATATATATATTTATTTGTTTACT
TTTTCTATCAGTGTTTTCAATTTTTTATTAAACAATGTTTGATTTTTTAAATCGCAA
TTTAATACCTAAATATAAAAAATGTTATTATATTGCAAAAACCCATCAACCTTGAA
AAAAAGTAGAAACGTTTTATTTAATTCTATCAATACATCATAAAATACGAACGATC
CCCGTCCAAGTTATGAGCTTAATCTTCCATAAAAATATTTGAAAGCAATAGATCA
TGTACTAAACTAAAATCAGGGAAATTcccggg

pCTL91 90bp-B2
gaattcCTCGATTTTTTTATGTTTAGTTTCGCGGACGACGGTTTCGAGGTGGCGGT
CTGGACCACGCCGGAGAGCGTCGAAGCGGAGGCGGTGTTCGCCGAGATCGG
CTCGCGCATGGCCGAGTTCAAAAGGCCTGCAGGCAAGTGCACAAACAATACTT
AAATAAATACTACTCAGTAATAACCTATTTCTTAGCATTTTTGACGAAATTCCGTC

GGCGTCTCGCACGACCACCAGTGCAAGGGTCTGAGCAGCGCCGTCGTGCTCC
TCGGAGTGGAGGCAGCCGAGCGCGACGGTGTGCCCGCCTTCCTGGAGACCTC
CGCGCTCCGCAACCTCCACTTCTACGAGCGGCTCGGCTTCACCGTCACCGCCG
ACGTCGAGGTGCCCGAAGGACCGCGCACCTGGTGCATGACCCGCAAGCCCGG
TGCCTGACGCTCGCCACACGACCCGCAGCGCCCGACCGAAAGGAGCGCACGA
cccggg

pCTL93 300bp[*lacO*]
gaattcCTCGATTTTTTTTATGTTTAGTTTCGCGGACGACGGTTTCGAGGTGGCGGT
CTGGACCACGCCGGAGAGCGTCGAAGCGGAGGCGGTGTTCGCCGAGATCGG
GAGCTCTCACACCTACAAGGGATGTACATCAATTGTGAGCGGATAACAATTGTT
AGGGAGGAATTGTGAGCGGATAACAATTTGGAGTTGATAATTGTGAGCGGATAA
CAATTGGCTTCAACGTAATTGTGAGCGGATAACAATTTCCGTACGAATGTGCCG
AACTTATGGTACCGCCGAGCGCGACGGTGTGCCCGCCTTCCTGGAGACCTCC
GCGCTCCGCAACCTCCACTTCTAAAAGCGGCTCGGCTTCAACTAAACATAAAAA
tACGAGGTGCCCGAAGGACCGCGCACCTGGTGCATGACCCGCAAGCCCGGTG
CCTGACGCTCGCCACACGACCCGCAGCGCCCGACCGAAAGGAGCGCACGAccc
ggg

pCTL94 One site[*lacO*]
gaattcCTCGATTTTTTTTATGTTTAGTTTCGCGGACGACGGTTTCGAGGTGGCGGT
CTGGACCACGCCGGAGAGCGTCGAAGCGGAGGCGGTGTTCGCCGAGATCGG
GAGCTCTCACACCTACAAGGGATGTACATCAATTGTGAGCGGATAACAATTGTT
AGGGAGGAATTGTGAGCGGATAACAATTTGGAGTTGATAATTGTGAGCGGATAA
CAATTGGCTTCAACGTAATTGTGAGCGGATAACAATTTCCGTACGAATGTGCCG
AACTTATGGTACCGCCGAGCGCGACGGTGTGCCCGCCTTCCTGGAGACCTCC
GCGCTCCGCAACCTCCACTTCTACGAGCGGCTCGGCTTCACCGTCACCGCCGA
CGTCGAGGTGCCCGAAGGACCGCGCACCTGGTGCATGACCCGCAAGCCCGGT
GCCTGACGCTCGCCACACGACCCGCAGCGCCCGACCGAAAGGAGCGCACGAc
ccggg

pTM0005 N-ARS1-N
ccgccctggagaatcGcggtgccgaggccgctcaattggtcgtagacagctctagcaccgcttaaacgcacgtacg
cgctgtcccccgcgttttaaccgccaaggggattactccctagtctccaggcacgtgtcagatatatacatcctgtATT
TTACAGATTTTATGTTTAGATCTTTTATGCTTGCTTTTCAAAAGGCCTGCAGGCA
AGTGCACAAACAATACTTAAATAAATACTACTCAGTAATAACCTATTTCTTAGCAT
TTTTGACGAAATTTGCTATTTTCCCAGTTCGCGCGCCCACCTACCGTGTGAAGT
CGTCACTCGGGCTTCTAAGTACGCTTAGCGCACGGTAGAGCGCAATCCAAGGC
TAACCACCGTGCATCGATGTTGAAAGAGGCCCTCCGTCCTTATTACTTCAAGTC
CCTGGGGTgcagg

pTM0023 N-90bp-N
ccgccctggagaatcGcggtgccgaggccgctcaattggtcgtagacagctctagcaccgcttaaacgcacgtacg
cgctgtcccccgcgttttaaccgccaaggggattactccctagtctccaggcacgtgtcagatatatacatcctgtATC
TCGATTTTTTTTATGTTTAGTTTCGCGGACGACGGTTTCGAGGTGGCGGTCTGGACCACG
CCGGAGAGCGTGTTCCAGGCTGGCCACGCAGCGAAGCGGAGGCGGTGTTCGCCGAG
ATCGGCTCGCGCAAAGCCGAGTTGAGCGAACTAAACATAAAAATACAGCACCCAGTTC
GCGCGCCCACCTACCGTGTGAAGTCGTCACTCGGGCTTCTAAGTACGCTTAGC
GCACGGTAGAGCGCAATCCAAGGCTAACCACCGTGCATCGATGTTGAAAGAGG
CCCTCCGTCCTTATTACTTCAAGTCCCTGGGGTgcagg

pCTL68 ARS1-Abf1
catatgcggtgtgaaataccgcacagatgcgtaaggagaaaataccgcatcaggcgccattcgccattcaggctgc
gcaactgttgggaagggcgatcggtgcgggcctcttcgctattacgccagctggcgaaagggggatgtgctgcaag
gcgattaagttgggtaacTATTTCTTAGCATTTTTGACGAAATTgtaaaacgacggccagtgaattc
ATTTTACAGATTTTATGTTTAGATCTTTTATGCTTGCTTTTCAAAAGGCCTGCAGG
CAAGTGCACAAACAATACTTAAATAAATACTACTCAGTAATAACCTATTTCTTAGC
ATTTTTGACGAAATTTGCTATTTTGTTAGAGTCTTTTACACCATTTGTCTCCACAC
CTCCGCTTACATCAACACCAATAACGCCATTTAATCTAAGCGCATCACCAACATT
TTCTGGCGTCAGTCCACCAGCTAACATAAAATGTAAGCTTTCGGGGCTCTCTTG
CCTTCCAACCCAGTCAGAAATCGAGTTCCAATCCAAAAGTTCACCTGTCCCACC
TGCTTCTGAATCAAACAAGGGAATAAACGAATGAGGTTTCTGTGAAGCTGCACT
GAGTAGTATGTTGCAcccggggatcc

pCTL67 90bp-Abf1
catatgcggtgtgaaataccgcacagatgcgtaaggagaaaataccgcatcaggcgccattcgccattcaggctgc
gcaactgttgggaagggcgatcggtgcgggcctcttcgctattacgccagctggcgaaagggggatgtgctgcaag
gcgattaagttgggtaacgccagggtTATTTCTTAGCATTTTTGACGAAATTacggccagtgaattc
CTCGATTTTTTTATGTTTAGTTTCGCGGACGACGGTTTCGAGGTGGCGGTCTGG
ACCACGCCGGAGAGCGTCGAAGCGGAGGCGGTGTTCGCCGAGATCGGCTCGC
GCATGGCCGAGTTGAGCGGTTCAAAGCTGGCCACGCAGAACTAAACATAAAAAt
ACCTGGCGCCGCTATTTCTTAGCATTTTTGACGAAATTCTGGCCACCGTCGGCG
TCTCGCACGACCACCAGTGCAAGGGTCTGAGCAGCGCCGTCGTGCTCCTCGG
AGTGGAGGCAGCCGAGCGCGACGGTGTGCCCGCCTTCCTGGAGACCTCCGCG
CTCCGCAACCTCCACTTCTACGAGCGGCTCGGCTTCACCGTCACCGCCGACGT
CGAGGTGCCCGAAGGACCGCGCACCTGGTGCATGACCCGCAAGCCCGGTGCC
TGACGCTCGCCACACGACCCGCAGCGCCCGACCGAAAGGAGCGCACGAcccgg
ggatcc

pCTL69 ARS317-Abf1
catatgcggtgtgaaataccgcacagatgcgtaaggagaaaataccgcatcaggcgccattcgccattcaggctgc
gcaactgttgggaagggcgatcggtgcgggcctcttcgctattacgccagctggcgaaagggggatgtgctgcaag
gcgattaagttgggtaacTATTTCTTAGCATTTTTGACGAAATTgtaaaacgacggccagtgaattc
ACTAGTACTTAAAAAAACTGTAGTTTCAGTGCAAAAAAGTTTTAACATTACGTATC
TTGTACCCTTTTTATTGCATATAGAAAGGTCAAATAATCCTTCACATCATGAAATA
TAAGCTAAATCGCATTTCTTTTCGTCCACATTTGCAAACAAAACTTTTCAATAATA
ATTTTATAAATAGTATCAATATATATATATATATATATATTTATTTGTTTACTTTTTC
TATCAGTGTTTTCAATTTTTTATTAAACAATGTTTGATTTTTTAAATCGCAATTTAA
TACCTAAATATAAAAAATGTTATTATATTGCAAAAACCCATCAACCTTGAAAAAAA
GTAGAAACGTTTTATTTAATTCTATCAATACATCATAAAATACGAACGATCCCCG
TCCAAGTTATGAGCTTAATCTTCCATAAAAATATTTGAAAGCAATAGATCATGTA
CTAAACTAAAATCAGGGAAATTcccggggatcc

Orc6-4A (S106A, S116A, S123A, T146A)
ATGCAACAAGTTCAACACTGTGTTGCTGAAGTTTTGAGATTGGACCCACAAGAA
AAGCCAGACTGGTCTTCTGGTTACTTGAAGAAGTTGACTAACGCTACTTCTATTT
TGTACAACACTTCTTTGAACAAGGTTATGTTGAAGCAAGACGAAGAAGTTGCTA
GATGTCACATTTGTGCTTACATTGCTTCTCAAAAGATGAACGAAAAGCACATGC
CAGACTTGTGTTACTACATTGACTCTATTCCATTGGAACCAAAGAAGGCTAAGC
ACTTGATGAACTTGTTCAGACAATCTTTGTCTAACTCTGCTCCAATGAAGCAATT

CGCTTGGACTCCAGCTCCAAAGAAGAACAAGAGAGCTCCAGTTAAGAACGGTG
GTAGATTCACTTCTTCTGACCCAAAGGAATTGAGAAACCAATTGTTCGGTGCTC
CAACTAAGGTTAGAAAGTCTCAAAACAACGACTCTTTCGTTATTCCAGAATTGCC
ACCAATGCAAACTAACGAATCTCCATCTATTACTAGAAGAAAGTTGGCTTTCGAA
GAAGACGAAGACGAAGACGAAGAAGAACCAGGTAACGACGGTTTGTCTTTGAA
GTCTCACTCTAACAAGTCTATTACTGGTACTAGAAACGTTGACTCTGACGAATAC
GAAAACCACGAATCTGACCCAACTTCTGAAGAAGAACCATTGGGTGTTCAAGAA
TCTAGATCTGGTAGAACTAAGCAAAACAAGGCTGTTGGTAAGCCACAATCTGAA
TTGAAGACTGCTAAGGCTTTGAGAAAGAGAGGTAGAATTCCAAACTCTTTGTTG
GTTAAGAAGTACTGTAAGATGACTACTGAAGAAATTATTAGATTGTGTAACGACT
TCGAATTGCCAAGAGAAGTTGCTTACAAGATTGTTGACGAATACAACATTAACG
CTTCTAGATTGGTTTGTCCATGGCAATTGGTTTGTGGTTTGGTTTTGAACTGTAC
TTTCATTGTTTTCAACGAAAGAAGAAGAAAGGACCCAAGAATTGACCACTTCATT
GTTTCTAAGATGTGTTCTTTGATGTTGACTTCTAAGGTTGACGACGTTATTGAAT
GTGTTAAGTTGGTTAAGGAATTGATTATTGGTGAAAAGTGGTTCAGAGACTTGC
AAATTAGATACGACGACTTCGACGGTATTAGATACGACGAAATTATTTTCAGAAA
GTTGGGTTCTATGTTGCAAACTACTAACATTTTGGTTACTGACGACCAATACAAC
ATTTGGAAGAAGAGAATTGAAATGGACTTGGCTTTGACTGAACCATTGTAA

Orc2-N-terminal truncation (delete a.a. 2-235)
ATGGCTTCTTCTTTCTTGGACACTTTCGAAGGTTACTTCGACCAAAGAAAGATTG
TTAGAACTAACGCTAAGTCTAGACACACTATGTCTATGGCTCCAGACGTTACTA
GAGAAGAATTCTCTTTGGTTTCTAACTTCTTCAACGAAAACTTCCAAAAGAGACC
AAGACAAAAGTTGTTCGAAATTCAAAAGAAGATGTTCCCACAATACTGGTTCGAA
TTGACTCAAGGTTTCTCTTTGTTGTTCTACGGTGTTGGTTCTAAGAGAAACTTCT
TGGAAGAATTCGCTATTGACTACTTGTCTCCAAAGATTGCTTACTCTCAATTGGC
TTACGAAAACGAATTGCAACAAAACAAGCCAGTTAACTCTATTCCATGTTTGATT
TTGAACGGTTACAACCCATCTTGTAACTACAGAGACGTTTTCAAGGAAATTACTG
ACTTGTTGGTTCCAGCTGAATTGACTAGATCTGAAACTAAGTACTGGGGTAACC
ACGTTATTTTGCAAATTCAAAAGATGATTGACTTCTACAAGAACCAACCATTGGA
CATTAAGTTGATTTTGGTTGTTCACAACTTGGACGGTCCATCTATTAGAAAGAAC
ACTTTCCAAACTATGTTGTCTTTCTTGTCTGTTATTAGACAAATTGCTATTGTTGC
TTCTACTGACCACATTTACGCTCCATTGTTGTGGGACAACATGAAGGCTCAAAA
CTACAACTTCGTTTTCCACGACATTTCTAACTTCGAACCATCTACTGTTGAATCT
ACTTTCCAAGACGTTATGAAGATGGGTAAGTCTGACACTTCTTCTGGTGCTGAA
GGTGCTAAGTACGTTTTGCAATCTTTGACTGTTAACTCTAAGAAGATGTACAAGT
TGTTGATTGAAACTCAAATGCAAAACATGGGTAACTTGTCTGCTAACACTGGTC
CAAAGAGAGGTACTCAAAGAACTGGTGTTGAATTGAAGTTGTTCAACCACTTGT
GTGCTGCTGACTTCATTGCTTCTAACGAAATTGCTTTGAGATCTATGTTGAGAGA
ATTCATTGAACACAAGATGGCTAACATTACTAAGAACAACTCTGGTATGGAAATT
ATTTGGGTTCCATACACTTACGCTGAATTGGAAAAGTTGTTGAAGACTGTTTTGA
ACACTTTGTAA

Supplementary Table 1: Primers

Primers	Sequence	Note	Source	Company
GC227	Biotin-gctgcaatgataccgcgagacccac	Amplify 3 kb template	Coster and Diffley, 2017	Sigma
GC228	Biotin-gggccagatggtaagccctc	Amplify 3 kb template	Coster and Diffley, 2017	Sigma
GC117	Biotin-atgcttccggctcgtatgttg	Amplify 0.6 kb template	Coster and Diffley, 2017	Sigma

GC298	gatgtgctgcaaggcgattaag	Amplify 0.6 kb template	Coster and Diffley, 2017	Sigma
EACS.B1 F	attcCTCGATTTTTTTATGTTTAGTTTCGCGGACGACGGTTTCGAGGTGG	Competitor DNA	Coster and Diffley, 2017	Sigma
EACS.B1 R	CCACCTCGAAACCGTCGTCCGCGAAACTAAACATAAAAAAATCGAGgaat	Competitor DNA	Coster and Diffley, 2017	Sigma
NCP F	Desthiobitin – CGAtagaaCTCGGGccgccctggagaatcGcggtgccg	Amplify nucleosome template	Miller et al., 2020	IDT
NCP R	CctgcACCCCAGGGACTTGAAGTAATAAGGAC	Amplify nucleosome template	Miller et al., 2020	IDT
CTL38	ATCTTTTGAATTTGCAAAATAACGTGGTTACCCCAGTACTTAG	Insertion for ORC2ΔN	This study	Sigma
CTL39	GCAAATTCAAAAGATGATTGACTTCTACAAGAAC	Vector for ORC2ΔN	This study	Sigma
CTL40	TATAGTTTTTTCTCCTTGACGTTAAAGTATAGAGG	Vector for ORC2ΔN	This study	Sigma
CTL61	ATCAGTGTTTTCAATTTTTTATTAAACAATGTTTGATTTTTTAAATCGCAATTTAATACCTggtcacccggccagcg	Replace endogenous ARS317::kanMX6	This study	Sigma
CTL62	ATGATCTATTGCTTTCAAATATTTTTATGGAAGATTAAGCTCATAACTTGGACGGGGATCGTTTAAACTGGATGGCGGCGTTAG	Replace endogenous ARS317::kanMX6	This study	Sigma
CTL98	TGCTACTCATCCTAGTCCTGTTGCTGCCAAGCTATTTAATATCATGCACGAAAAGCAAACcgacatggaggcccag	Replace URA3::hphNT1	This study	IDT
CTL99	GGCCGCATCTTCTCAAATATGCTTCCCAGCCTGCTTTTCTGTAACGTTCACCCTCTACCTccttcgagcgtcccaaaac	Replace URA3::hphNT1	This study	IDT
CTL86	GCCTTTTATATACCGATTAAATATTTATATTTCTGACTTTTTCTTTACCGACACCAACaggcctgtttaaacGCTTTTCAATTCATCTTTTTTTTTTTTG	Insertion of URA3 in ARS419	This study	IDT
CTL87	TTCTATTCGTAAGAACTGTTTATAGAATACTCTTTTTTTTTATGTTTTGAGCCTACACCTtctagaaagcttcatatgTTAGTTTTGCTGGCCGCATC	Insertion of URA3 in ARS419	This study	IDT
CTL125	GGAGAAAAAACTATACACCGGTGTATAAAACAATGGCTTCTTCTTTCTTGGACACTTTCGAAGGTTACTTC	Insertion for ORC2ΔN	This study	IDT
CTL141	ggtgtgaaataccgcacagatg	qPCR detection site	This study	IDT
CTL142	gctggcgtaatagcgaagag	qPCR detection site	This study	IDT
CTL143	GTGCATGGCCTTCTTTTCTAATTG	Internal control for qPCR	This study	IDT
CTL144	GCAGGATTAGGTAGCGTTTAATTTTATCC	Internal control for qPCR	This study	IDT
CTL147	TATTTCTTAGCATTTTTGACGAAATTacggccagtgaattcCTCG	Insert for pCTL67	This study	IDT
CTL148	AATTTCGTCAAAAATGCTAAGAAATAGCGGCGCCAGGTaTTTTTATG	Insert for pCTL67	This study	IDT
CTL149	ATTTTTGACGAAATTCTGGCCACCGTCGGC	Vector for pCTL67	This study	IDT
CTL150	AAATGCTAAGAAATAaccctggcgttacccaac	Vector for pCTL67	This study	IDT
CTL156	TATTTCTTAGCATTTTTGACGAAATTacggccagtgaattcACTAGTAC	Insert for pCTL68 and pCTL69	This study	IDT
CTL157	AATTTCGTCAAAAATGCTAAGAAATActctagaggatccccggg	Insert for pCTL68 and pCTL69	This study	IDT
CTL158	AAATGCTAAGAAATAgttacccaacttaatcgccttgc	Vector for pCTL68 and pCTL69	This study	IDT
CTL159	ATTTTTGACGAAATTgtaatcatggtcatagctgtttcctg	Vector for pCTL68 and pCTL69	This study	IDT
CTL160	GCAGCTGGTGTTTTCATCGAACATGATGAGCTTTAAACAAGGAGGAGTATAAACTTTTAAcatatgcggtgtgaaataccgc	Amplify sequence for ARS419 insertion	This study	IDT
CTL161	ATAACCGATGACTTGGCAAGAAAAGGATTTTAAGAGTTGTTCTATTCGTAAGAACTGTTTcaggaaacagctatgaccatgattac	Amplify sequence for ARS419 insertion	This study	IDT
CTL172	TAGGTGTGAGAGCTCCCGATCTCGGCGAACAC	Amplify sequence for LacO plasmid	This study	IDT
CTL173	CGAACTTATGGTACCGCCGAGCGCGACG	Amplify sequence for LacO plasmid	This study	IDT
CTL174	GAGCTCTCACACCTACAAGGGATGTACATCAATTGTGAGCGGATAACAATTGTTAGGGAGGAATTGTGAGCGGATAACAATTTGGAGTTGATAATTGTGAGCGGATAACAATTGGCTTCAACGTAATTGTGAGCGGATAACAATTTCCGTACGAATGTGCCGAACTTATGGTACC	Duplex DNA of LacO	This study	IDT
CTL208	TCAAAAGGCCTGCAGGCAAGTGCACAAACAATACTTAAATAAATACTACTCAGTAATAACCTATTTCTTAGCATTTTTGACGAAATT	oligo for ARS1_B2	This study	IDT
CTL209	AATTTCGTCAAAAATGCTAAGAAATAGGTTATTACTGAGTAGTATTTATTTAAGTATTGTTTGTGCACTTGCCTGCAGGCCTTTTGA	oligo for ARS1_B2	This study	IDT
CTL214	CTTGCCTGCAGGCCTTTTGAACTCGGCCATGCGC	Amplify sequence for 90bp-B2	This study	IDT
CTL215	TTAGCATTTTTGACGAAATTCCGTCGGCGTCTCG	Amplify sequence for 90bp-B2	This study	IDT
50bp up 209	TTAAGTTGGGTAACGCCAGGG	Amplify sequence for EMSA	This study	Sigma
50bp down 209	CCAGCTGAGGTACCGGATGCT	Amplify sequence for EMSA	This study	Sigma

Supplementary Table 2: Plasmids

Plasmid number	Plasmid	Vector	Insert source	Insert sites	Source
pGC199	No site	pUC19			From Coster *et al.*, Science 2017.
pGC200	One site	pUC19			From Coster *et al.*, Science 2017.
pGC201	25bp	pUC19			From Coster *et al.*, Science 2017.

pGC202	50bp	pUC19			From Coster *et al.*, Science 2017.
pGC211	90bp	pUC19			From Coster *et al.*, Science 2017.
pCTL8	pRS304-Orc5/Orc6-4A	pJF18	synthetic GeneArt strings (Thermo Fisher Scientific)	BlpI/EcoRI	pJF18 from Frigola *et al*, Nature 2013.
pCTL9	12bp	pUC19	synthetic GeneArt strings (Thermo Fisher Scientific)	BsoBI/EcoRI	This study
pCTL40	10bp	pUC19	synthetic GeneArt strings (Thermo Fisher Scientific)	BsoBI/EcoRI	This study
pCTL64	pRS306-Orc1/Orc2ΔN	PCR from pJF19 with CTL39 and CTL40	PCR from pJF19 with CTL125 and CTL38	In-fusion	pJF19 from Frigola *et al*, Nature 2013.
pCTL67	pUC19-90bp-Abf1	PCR from pGC211 with CTL149 and CTL150	PCR from pGC211 with CTL147 and CTL148	In-fusion	pGC211 from Coster *et al.*, Science 2017.
pCTL68	pUC19-ARS1-Abf1	PCR from pGC403 with CTL158 and CTL159	PCR from pGC403 with CTL156 and CTL157	In-fusion	pGC403 from Coster *et al.*, Science 2017.
pCTL69	pUC19-ARS317-Abf1	PCR from pCTL45 with CTL158 and CTL159	PCR from pCTL45 with CTL156 and CTL157	In-fusion	This study
pCTL86	pUC19-ARS317	pUC19	synthetic GeneArt strings 495bp (Thermo Fisher Scientific)	EcoRI/BsoBI	This study
pCTL91	pUC19-90bp-B2	pGC211	PCR pGC211 with primer CTL214 and CTL215; ARS1_B2 sequence generated from IDT as oligo CTL208 and CTL209	In-fusion	pGC211 from Coster *et al.*, Science 2017.
pCTL93	pUC19-300bp+LacO	pGC205	PCR pGC200 with primer CTL172 and CTL173; LacO sequence generated from IDT as duplex DNA CTL174	In-fusion	pGC 205 from Coster *et al.*, Science 2017.
pCTL94	pUC-One site+LacO	pGC200	PCR pGC200 with primer CTL172 and CTL173; LacO sequence generated from IDT as duplex DNA CTL174	In-fusion	pGC200 from Coster *et al.*, Science 2017.

Supplementary Table 3: Yeast strains

**Yeast strain**	**Background**	**Genotype**	**Source**	
ySDORC	W303-1a	*MATa ade2-1 ura3-1 his3-11,15 trp1-1 leu2-3,112 can1-100*<br>*bar1::Hyg*<br>*pep4::KanMx*<br>*his3::pRS303-Gal1,10-ORC3/4 (HIS3)*<br>*ura3::pRS306-Gal1,10-ORC1/2 CBP-Orc1 (URA3)*<br>*trp1::pRS304-Gal1,10-ORC5/6 (TRP1)*<br>*MATa ade2-1 ura3-1 his3-11,15 trp1-1 leu2-3,112 can1-100*<br>*bar1::Hyg*<br>*pep4::KanMx*	Frigola *et al*, Nature 2013.	
YGC229	W303-1a	*his3::pRS303-Gal1,10-Cdt1/Gal4 (HIS3)*<br>*ura3::pRS306-Gal1,10-Mcm2/3 (URA3) CBP-TEV-NanoLuc-Mcm3*<br>*trp1::pRS304-Gal1,10-Mcm4/5 (TRP1)*<br>*leu2::pRS305-Gal1,10-Mcm6/7 (LEU2)*<br>*MATa ade2-1 ura3-1 his3-11,15 trp1-1 leu2-3,112 can1-100*<br>*bar1::Hyg*<br>*pep4::KanMx*	Coster *et al.*, Science 2017.	
yCTL6<br>(Orc6-4A)	W303-1a	MATa ade2-1 ura3-1 his3-11,15 trp1-1 leu2-3,112 can1-100<br>bar1::Hyg<br>pep4::KanMX<br>Flag at WT ORC6 (Leu) c-terminal<br>ura::URApRS306/SDORC1,2<br>his::HISpRS303/SDORC3,4<br>trp::TRPpRS304/SDORC5,ORC6-4S/T-A	This study	
yCTL17<br>(Orc2_deltaN)	W303-1a	MATa ade2-1 ura3-1 his3-11,15 trp1-1 leu2-3,112 can1-100<br>bar1::Hyg<br>pep4::KanMX<br>Flag at WT ORC2 (Nat) C-terminal<br>ura::URApRS306/SDORC1,ORC2_del2-235<br>his::HISpRS303/SDORC3,4<br>trp::TRPpRS304/SDORC5,ORC6	This study	RE digestion with NcoI on pCTL64
yCTL28<br>(ARS1Δ)	A364a	MATa ade2 ade3 ura3-52 trp1-289 leu2-3,112<br>bar1::LEU2<br>ORC2<br>ORC6<br>ura3-52::GAL1p-Δntcdc6, URA3 ΔURA3::hph<br>cdc20::MET3p-HA3-CDC20, TRP1<br>MCM7-2NLS<br>ARS317::KanMX6 ΔARS419::ARS1Δ	This study	PCR on pGC404 with CTL160 and CTL161.<br>pGC404 from Coster *et al.*, Science 2017.
yCTL29<br>(ARS1)	A364a	MATa ade2 ade3 ura3-52 trp1-289 leu2-3,112<br>bar1::LEU2<br>ORC2<br>ORC6<br>ura3-52::GAL1p-Δntcdc6, URA3 ΔURA3::hph	This study	PCR on pCTL68 with CTL160 and CTL161

		cdc20::MET3p-HA3-CDC20, TRP1<br>MCM7-2NLS<br>ARS317::KanMX6 ∆ARS419::ARS1		
yCTL30<br>(ARS317)	A364a	MATa ade2 ade3 ura3-52 trp1-289 leu2-3,112<br>bar1::LEU2<br>ORC2<br>ORC6<br>ura3-52::GAL1p-∆ntcdc6, URA3 ∆URA3::hph<br>cdc20::MET3p-HA3-CDC20, TRP1<br>MCM7-2NLS<br>ARS317::KanMX6 ∆ARS419::ARS317	This study	PCR on pCTL69 with CTL160 and CTL161
yCTL33<br>(90bp)	A364a	MATa ade2 ade3 ura3-52 trp1-289 leu2-3,112<br>bar1::LEU2<br>ORC2<br>ORC6<br>ura3-52::GAL1p-∆ntcdc6, URA3 ∆URA3::hph<br>cdc20::MET3p-HA3-CDC20, TRP1<br>MCM7-2NLS<br>ARS317::KanMX6 ∆ARS419::90bp	This study	PCR on pCTL67 with CTL160 and CTL161
