# Supplementary material for: Cell cycle regulation has shaped replication origins in budding yeast
Source: Nat Struct Mol Biol. 2025 Jun 30;32(9):1697–707. doi: 10.1038/s41594-025-01591-9 (PMC12440816; doi:10.1038/s41594-025-01591-9)
Supplement: Supplementary file 2 — Reporting Summary [file 41594_2025_1591_MOESM2_ESM.pdf]

nature portfolio

Corresponding author(s): Thomas Miller and John Diffley

Last updated by author(s): Jul 8, 2024

Reporting Summary

Nature Portfolio wishes to improve the reproducibility of the work that we publish. This form provides structure for consistency and transparency in reporting. For further information on Nature Portfolio policies, see our Editorial Policies and the Editorial Policy Checklist.

Statistics

For all statistical analyses, confirm that the following items are present in the figure legend, table legend, main text, or Methods section.

n/a	Confirmed	
☐	☒	The exact sample size (*n*) for each experimental group/condition, given as a discrete number and unit of measurement
☐	☒	A statement on whether measurements were taken from distinct samples or whether the same sample was measured repeatedly
☐	☒	The statistical test(s) used AND whether they are one- or two-sided<br>*Only common tests should be described solely by name; describe more complex techniques in the Methods section.*
☒	☐	A description of all covariates tested
☒	☐	A description of any assumptions or corrections, such as tests of normality and adjustment for multiple comparisons
☐	☒	A full description of the statistical parameters including central tendency (e.g. means) or other basic estimates (e.g. regression coefficient) AND variation (e.g. standard deviation) or associated estimates of uncertainty (e.g. confidence intervals)
☐	☒	For null hypothesis testing, the test statistic (e.g. *F*, *t*, *r*) with confidence intervals, effect sizes, degrees of freedom and *P* value noted<br>*Give P values as exact values whenever suitable.*
☒	☐	For Bayesian analysis, information on the choice of priors and Markov chain Monte Carlo settings
☒	☐	For hierarchical and complex designs, identification of the appropriate level for tests and full reporting of outcomes
☒	☐	Estimates of effect sizes (e.g. Cohen's *d*, Pearson's *r*), indicating how they were calculated

Our web collection on statistics for biologists contains articles on many of the points above.

Software and code

Policy information about availability of computer code

Data collection	Typhoon phosphorimager (GE Healthcare), BMG LABTECH's Reader Control software
Data analysis	Prism 10, ImageJ(Version 1.53), Snapgene (Version 8.0), Adobe Photoshop 2024

For manuscripts utilizing custom algorithms or software that are central to the research but not yet described in published literature, software must be made available to editors and reviewers. We strongly encourage code deposition in a community repository (e.g. GitHub). See the Nature Portfolio guidelines for submitting code & software for further information.

Data

Policy information about availability of data

All manuscripts must include a data availability statement. This statement should provide the following information, where applicable:

- Accession codes, unique identifiers, or web links for publicly available datasets
- A description of any restrictions on data availability
- For clinical datasets or third party data, please ensure that the statement adheres to our policy

The data supporting this study are available within the paper, its Supplementary Information and Source Data files. Atomic model coordinates and cryo-EM maps have been deposited in the Protein Data Bank (PDB) and Electron Microscopy Data Bank (EMDB), under the accession codes 8RIF/EMD-19186 (DH), 8RIG/EMD-19187 (SH), and 9I3I (MO with Orc2-IDR). Access to unprocessed electron microscopy datasets can be provided by the corresponding author upon reasonable request.

Research involving human participants, their data, or biological material

Policy information about studies with human participants or human data. See also policy information about sex, gender (identity/presentation), and sexual orientation and race, ethnicity and racism.

Reporting on sex and gender	n/a
Reporting on race, ethnicity, or other socially relevant groupings	n/a
Population characteristics	n/a
Recruitment	n/a
Ethics oversight	n/a

Note that full information on the approval of the study protocol must also be provided in the manuscript.

Field-specific reporting

Please select the one below that is the best fit for your research. If you are not sure, read the appropriate sections before making your selection.

☒ Life sciences ☐ Behavioural & social sciences ☐ Ecological, evolutionary & environmental sciences

For a reference copy of the document with all sections, see nature.com/documents/nr-reporting-summary-flat.pdf

Life sciences study design

All studies must disclose on these points even when the disclosure is negative.

Sample size	Each MCM loading/recruitment assays were in triplicates for technical repeat. For in vivo-re-replication assay, three biological repeat samples were collected and repeated twice (for high and low Ct values) with triplicates. Sample sizes was determined based on statistical consideration.
Data exclusions	No data was excluded
Replication	Each MCM loading assays, recruitment assays, in vitro replication assays were individually repeated at least twice, all replicates were successful.<br>In vivo re-replication was repeated in three biological repeats, the experiments were performed multiple times; however only complete data sets were elected due to technical issues, primarily pipetting errors, affecting result accuracy and reliability.<br>Each negative stain EM experiment was repeated at least twice, with multiple time-points.
Randomization	Biochemical assays do not involve randomization because they follow a predefined experimental protocol where specific reagents and conditions are carefully controlled. Biochemical assays focus on precise and reproducible measurement under set conditions.
Blinding	Blinding is not applicable to biochemical assays because these experiments follow a standardized protocol.

Reporting for specific materials, systems and methods

We require information from authors about some types of materials, experimental systems and methods used in many studies. Here, indicate whether each material, system or method listed is relevant to your study. If you are not sure if a list item applies to your research, read the appropriate section before selecting a response.

Materials & experimental systems

n/a	Involved in the study	
☐	☒	Antibodies
☒	☐	Eukaryotic cell lines
☒	☐	Palaeontology and archaeology
☒	☐	Animals and other organisms
☒	☐	Clinical data
☒	☐	Dual use research of concern
☒	☐	Plants

Methods

n/a	Involved in the study	
☒	☐	ChIP-seq
☒	☐	Flow cytometry
☒	☐	MRI-based neuroimaging

Antibodies

Antibodies used	Polyclonal antibody JDI 70, produced in rabbit against yeast Cdt1, polyclonal antibody E78, produced in rabbit against yeast Mcm6
Validation	JDI 70 antibody is cited in Drury, L. S. & Diffley, J. F. X. Factors affecting the diversity of DNA replication licensing control in eukaryotes. Curr Biol 19, 530-535 (2009). E78 antibody data is provided in this manuscript.

Plants

Seed stocks	*Report on the source of all seed stocks or other plant material used. If applicable, state the seed stock centre and catalogue number. If plant specimens were collected from the field, describe the collection location, date and sampling procedures.*
Novel plant genotypes	*Describe the methods by which all novel plant genotypes were produced. This includes those generated by transgenic approaches, gene editing, chemical/radiation-based mutagenesis and hybridization. For transgenic lines, describe the transformation method, the number of independent lines analyzed and the generation upon which experiments were performed. For gene-edited lines, describe the editor used, the endogenous sequence targeted for editing, the targeting guide RNA sequence (if applicable) and how the editor was applied.*
Authentication	*Describe any authentication procedures for each seed stock used or novel genotype generated. Describe any experiments used to assess the effect of a mutation and, where applicable, how potential secondary effects (e.g. second site T-DNA insertions, mosiacism, off-target gene editing) were examined.*
