# Supplementary figures and images for: Cell cycle regulation has shaped replication origins in budding yeast
Source: Nat Struct Mol Biol. 2025 Jun 30;32(9):1697–707. doi: 10.1038/s41594-025-01591-9 (PMC12440816; doi:10.1038/s41594-025-01591-9)

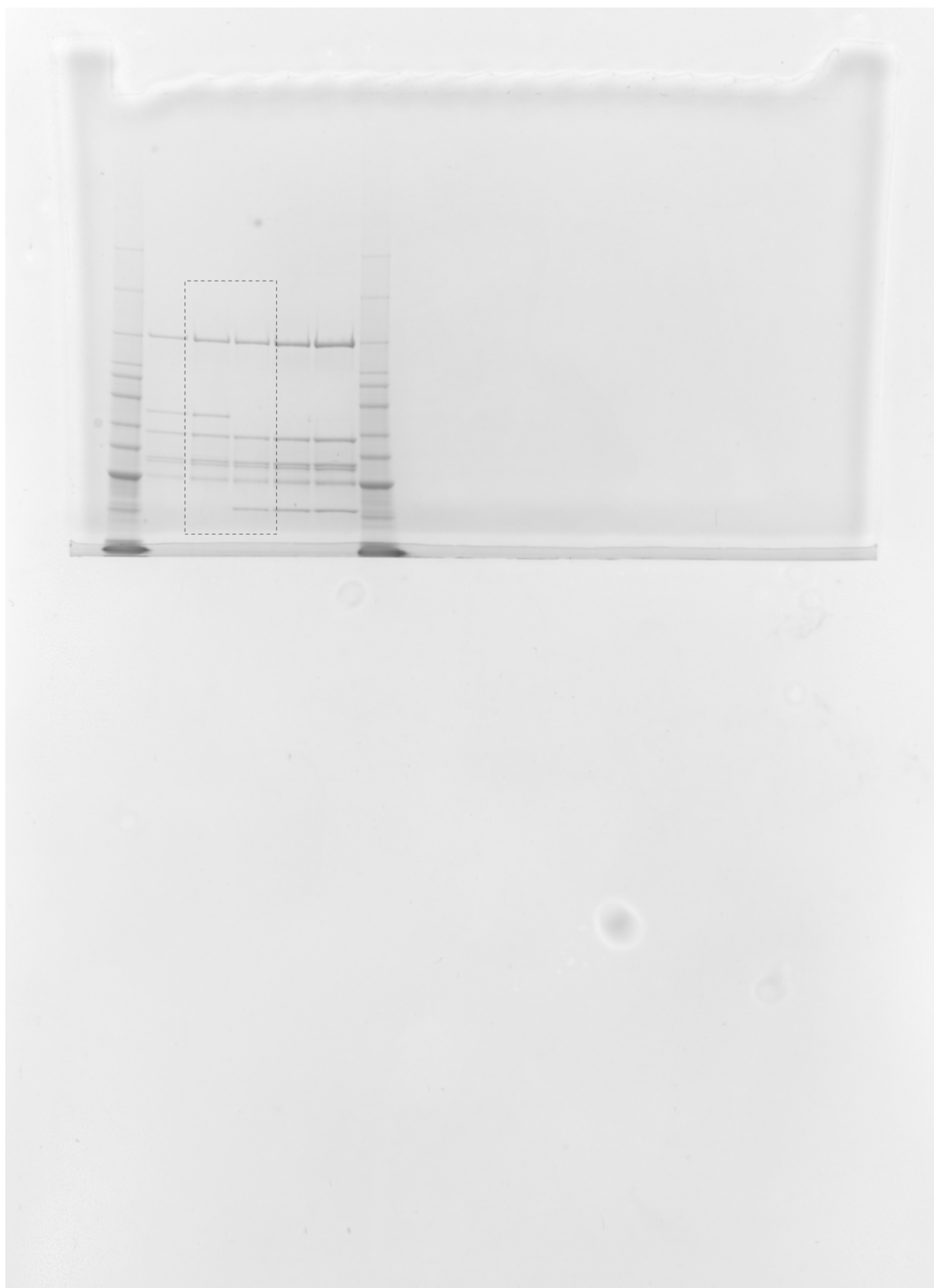

Supplement: Supplementary file 5 — Fig. 1d: unprocessed gel. [file 41594_2025_1591_MOESM5_ESM.pdf]

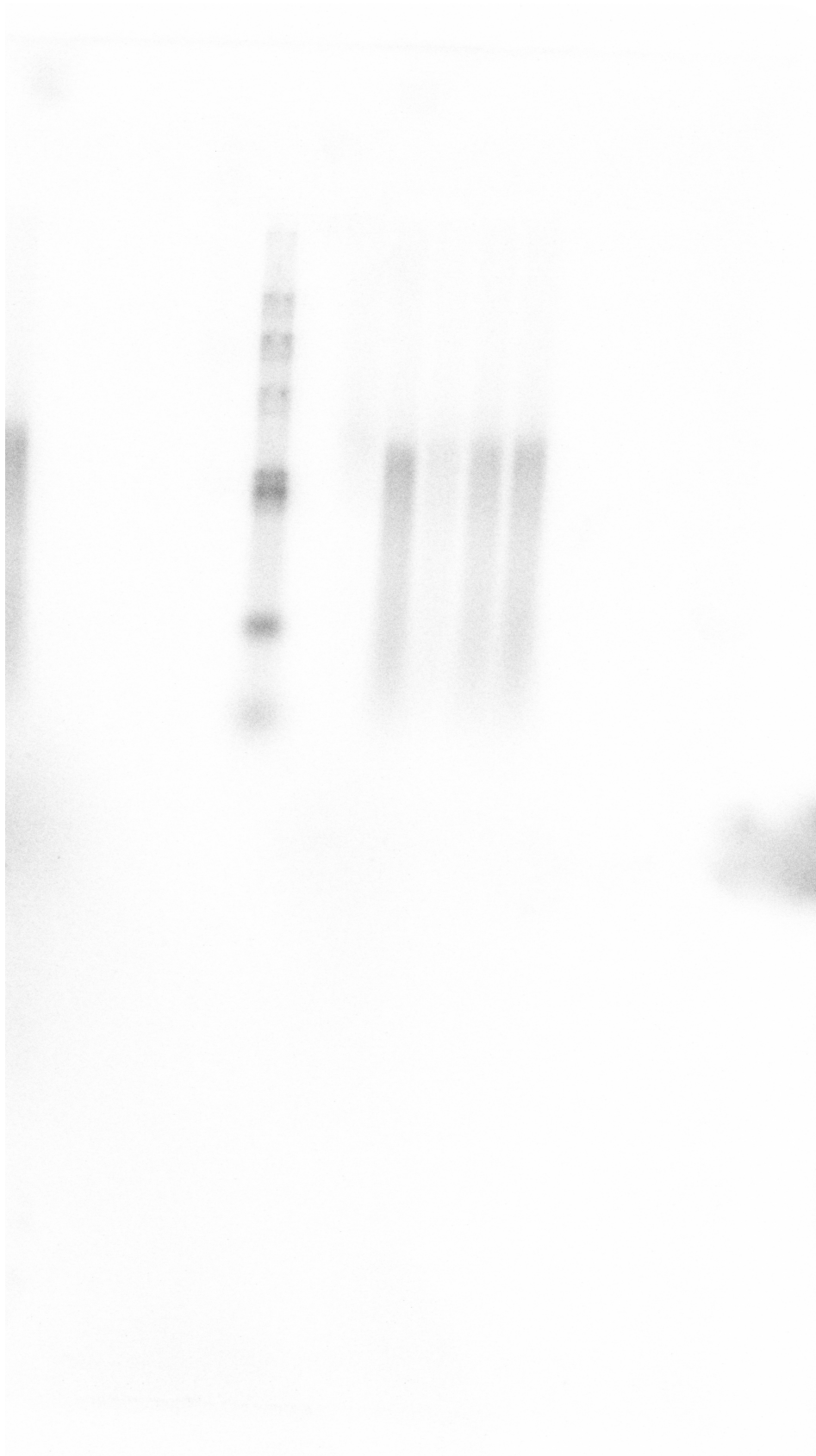

Supplement: Supplementary file 8 — Fig. 2d: Unprocessed gel. [file 41594_2025_1591_MOESM8_ESM.pdf]

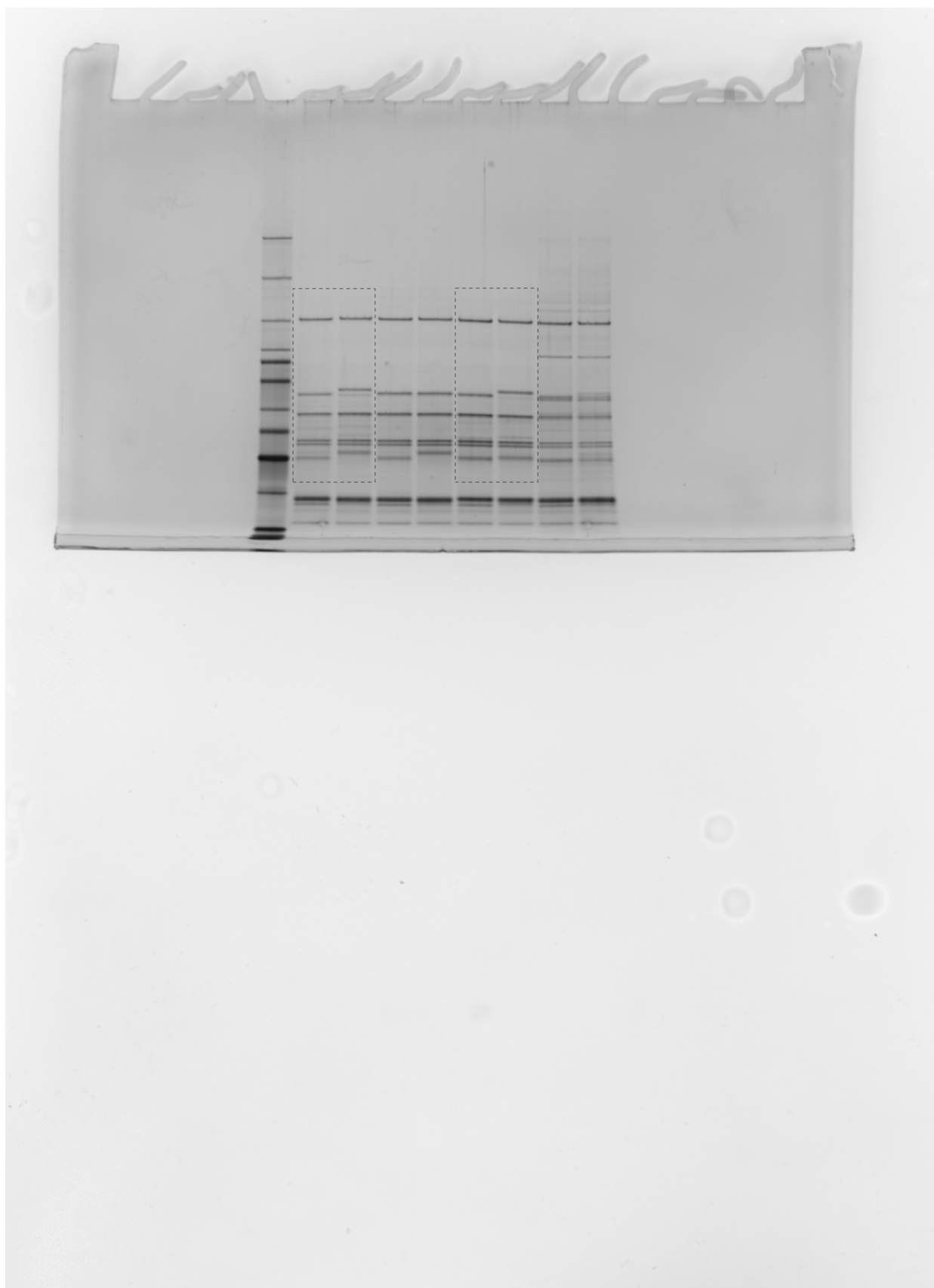

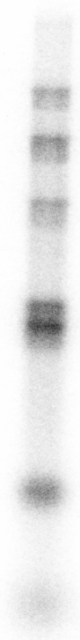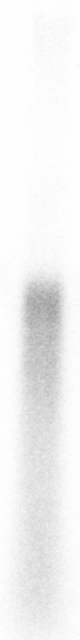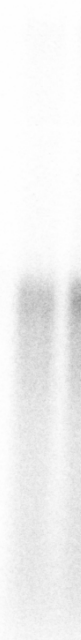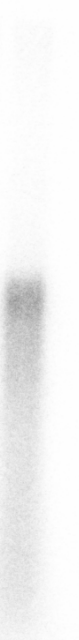

Supplement: Supplementary file 10 — Fig. 3a,c: unprocessed gel; Fig. 3b(ii), Fig. 3f(i) and Fig. 3g: negative-stain EM. [file 41594_2025_1591_MOESM10_ESM.pdf]

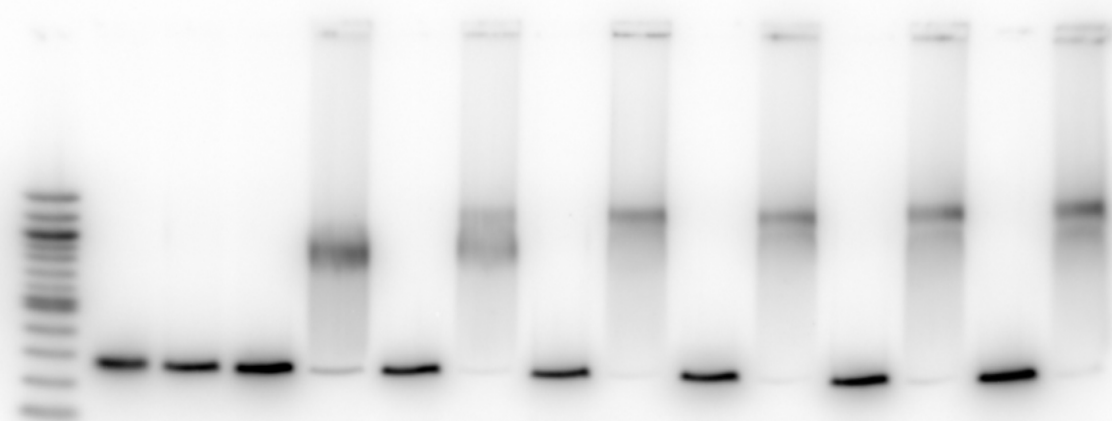

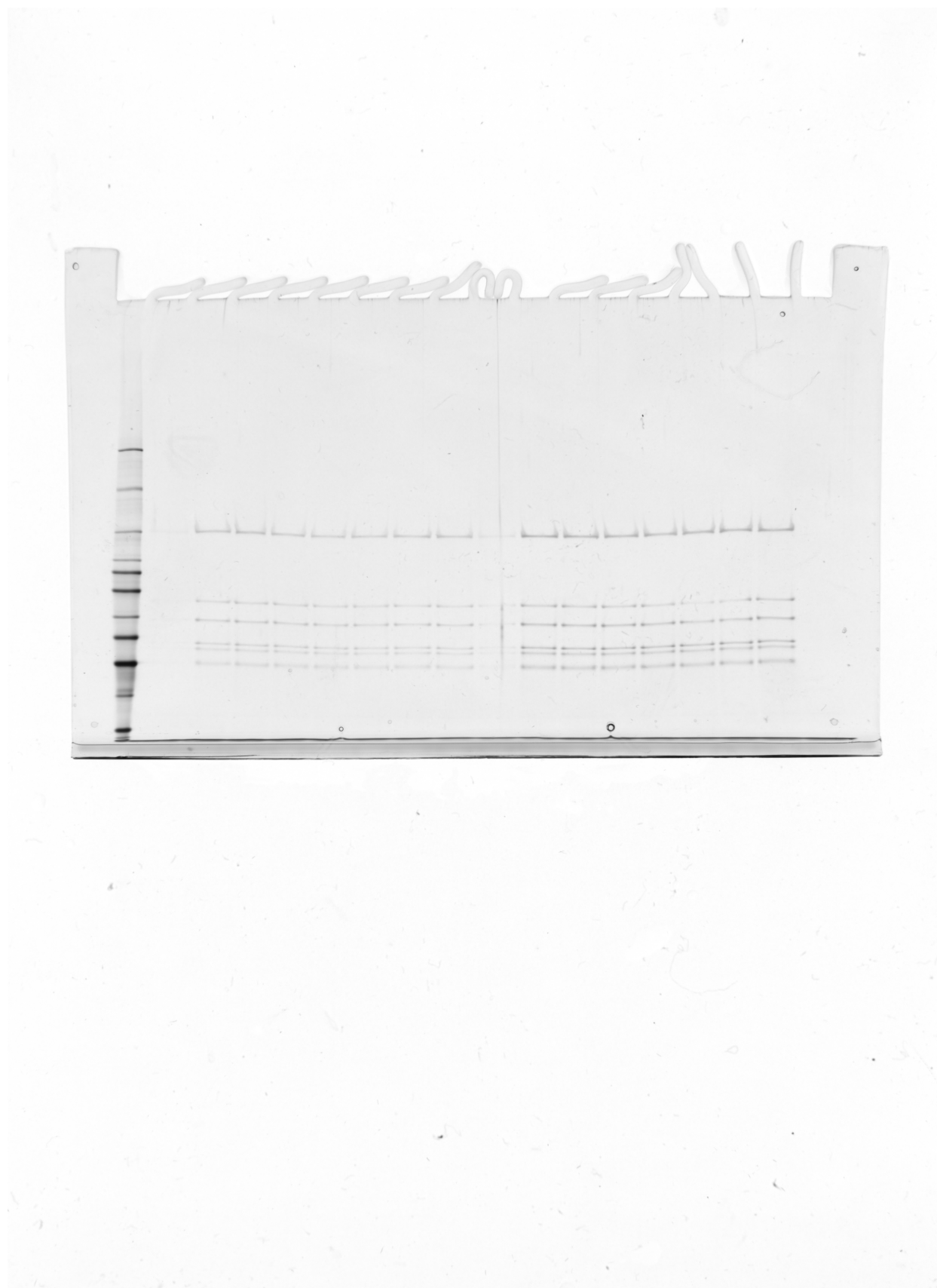

Supplement: Supplementary file 13 — ED_Fig. 2a,d: unprocessed gel. [file 41594_2025_1591_MOESM13_ESM.pdf]

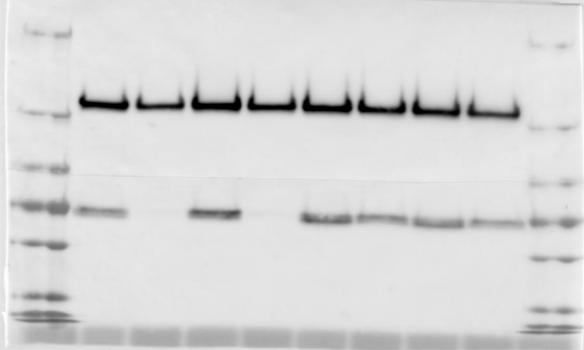

Supplement: Supplementary file 15 — ED_Fig. 6c: unprocessed western blot. [file 41594_2025_1591_MOESM15_ESM.pdf]
